# Genome-Scale Analysis of Translation Elongation with a Ribosome Flow Model

**DOI:** 10.1371/journal.pcbi.1002127

**Published:** 2011-09-01

**Authors:** Shlomi Reuveni, Isaac Meilijson, Martin Kupiec, Eytan Ruppin, Tamir Tuller

**Affiliations:** 1 Department of Statistics and Operations Research, School of Mathematical Sciences, Tel Aviv University, Ramat Aviv, Israel; 2 School of Chemistry, Tel Aviv University, Ramat Aviv, Israel; 3 Molecular Microbiology and Biotechnology, Tel Aviv University, Ramat Aviv, Israel; 4 School of Computer Sciences, Tel Aviv University, Ramat Aviv, Israel; 5 School of Medicine, Tel Aviv University, Ramat Aviv, Israel; 6 Faculty of Mathematics and Computer Science, Weizmann Institute of Science, Rehovot, Israel; 7 Department of Molecular Genetics, Weizmann Institute of Science, Rehovot, Israel; University of British Columbia, Canada

## Abstract

We describe the first large scale analysis of gene translation that is based on a model that takes into account the physical and dynamical nature of this process. The Ribosomal Flow Model (*RFM*) predicts fundamental features of the translation process, including translation rates, protein abundance levels, ribosomal densities and the relation between all these variables, better than alternative (‘non-physical’) approaches. In addition, we show that the *RFM* can be used for accurate inference of various other quantities including genes' initiation rates and translation costs. These quantities could not be inferred by previous predictors. We find that increasing the number of available ribosomes (or equivalently the initiation rate) increases the genomic translation rate and the mean ribosome density only up to a certain point, beyond which both saturate. Strikingly, assuming that the translation system is tuned to work at the pre-saturation point maximizes the predictive power of the model with respect to experimental data. This result suggests that in all organisms that were analyzed (from bacteria to Human), the global initiation rate is optimized to attain the pre-saturation point. The fact that similar results were *not* observed for heterologous genes indicates that this feature is under selection. Remarkably, the gap between the performance of the *RFM* and alternative predictors is strikingly large in the case of heterologous genes, testifying to the model's promising biotechnological value in predicting the abundance of heterologous proteins before expressing them in the desired host.

## Author Summary

Gene translation is a central process in all living organisms. However, this process is still enigmatic, and contradicting conclusions regarding the essential parameters that determine translation rates appear in different studies. We introduce a new approach for modeling the process of translation elongation. Taking into account the stochastic nature of the translation process and the excluded volume interactions between ribosomes, our model is aimed at capturing the effect of codon order and composition on translation rates. We demonstrate that in comparison to commonly used approaches, our approach gives more accurate predictions of translation rates, protein abundance levels and ribosome densities across many species. Using our model, we address the need for a better understanding of the inner workings of the translation process. To this end, we analyze large scale genomic measurements made in several organisms. Our analysis unravels several central and previously uncharacterized aspects of the translation process. For example, we show that in all organisms that were analyzed (from bacteria to Human), ribosome allocation is optimized to give maximal translation rate in minimal cost. In addition, we provide the first direct estimate for the effect of codon order on protein abundance, showing that in E. coli and S. cerevisiae it can solely account for more than 20% of the variation in this quantity.

## Introduction

Gene translation is a complex process through which an mRNA sequence is decoded by the ribosome to produce a specific protein. The elongation step of this process is an iterative procedure in which each codon in the mRNA sequence is recognized by a specific tRNA, which adds one additional amino-acid to the growing peptide [1]. As gene translation is a central process in all living organisms, its understanding has ramifications to human health [2], [3], [4], biotechnology [5], [6], [7], [8], [9], [10], [11], [12] and evolution [4], [7], [11], [13].

In recent years there has been a sharp growth in the number of new technologies for measuring different features related to the process of gene translation [5], [6], [10], [14], [15], [16], [17], [18], [19]. However, this process is still enigmatic, with contradicting conclusions in different studies. In particular, the identity of the essential parameters that determine translation rates is still under debate [6], [20], [21]. Recent studies have suggested that the order of codons along the mRNA (and not only the composition of codons) plays an important role in determining translation efficiency [7], [20], [22], [23]. Starting with the seminal work of MacDonald et al. [24], [25] and the work of Heinrich et al. [24], [25] theoretical models for the movement of ribosomes (and other biological ‘machines’) have been presented [26], [27], [28]. Despite being relatively realistic these models haven't been used for the analysis of large scale genomic data. The models that have been used for this purpose, while making promising and worthy first strides, have not attempted to capture the nature of the translation elongation process on all its various physical aspects [6], [13], [26], [27], [28], [29], [30].

The most widely used predictors of translation efficiency are the codon adaptation index (CAI) [28] and the tRNA adaptation index (tAI) [27]. As we describe later, the tAI is the mean adaptation of a gene (i.e., of its codons) to the tRNA pool of the organism. The CAI is similar to the tAI albeit in this predictor the weight of each codon is computed based on its frequency in a set of highly expressed genes. Based on measures such as the tAI, it is possible to estimate the translation rate of single codons. Thus, it possible to study (local) translation rate profiles along genes [7], [31]. As we depict later, in this study we take into account some additional physical aspects of translation elongation.

The aim of the present research is twofold:

First, we address the need for a simple, physically plausible computational model that is *solely based on the coding sequence* (*i.e.* a vector of codons in each gene). In addition we further require that the model will allow for a computationally efficient analysis of the translation process on a genome-wide scale and across many species. Focusing on the coding sequence, we by no means wish to imply that it is only factor taking place in the determination of translation rates. Nevertheless, since it has been widely recognized as a prime factor in the translation elongation process, we will herby study it in isolation. To this end, we introduce a new approach for modeling translation elongation. Our model is aimed at capturing the effect of codon order on translation rates, the stochastic nature of the translation process and the interactions between ribosomes. We demonstrate that our approach gives more accurate predictions of translation rates, protein abundance and ribosome densities in endogenous and heterologous genes in comparison to contemporary approaches.

Second, using our model, we address the need for a better understanding of the translation process. Our analysis unravels several central and yet uncharacterized aspects of this process.

## Results

### A Stochastic Flow Model of Translation Elongation

**Figure 1:**
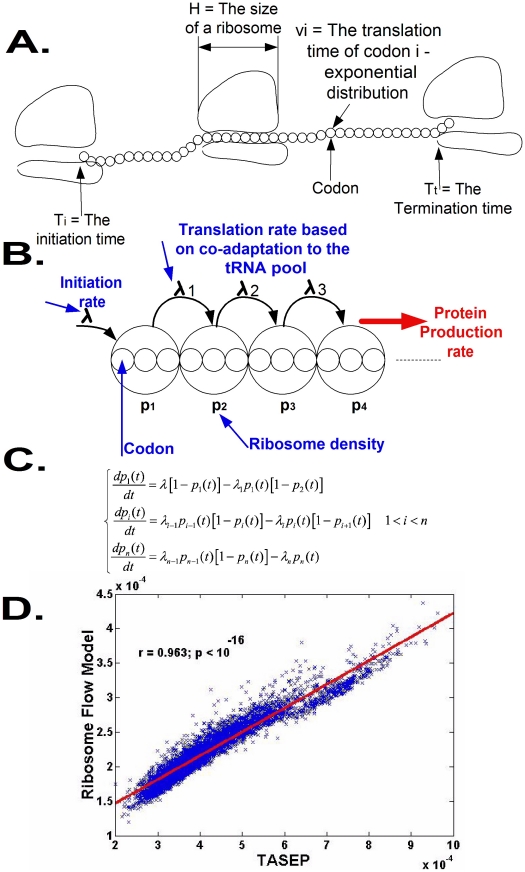
Basic properties of the Ribosome Flow Model (RFM). A. The TASEP model: each codon has an exponentially distributed translation time; ribosomes have volume and can block each other. B. The *RFM has* two free parameters: the initiation rate λ and the number of codons *C* at each ‘site’ (proportional to the size of the ribosome). Each site has a corresponding transition rate 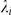 that is estimated based on the co-adaptation between the codons of the site and the tRNA pool of the organism (Methods). The output of the model consists of the steady state occupancy probabilities of ribosomes at each site and the steady state translation rates, or ribosome flow through the system. C. The set of differential equations that describe the RFM, denoted as equation (1). D. *RFM* vs. TASEP: the correlation between translation rates predicted by the two models is close to perfect (r = 0.963, p<10^−16^) while the running time of the TASEP is *orders of magnitude* longer (usually several days *vs.* minutes).

Our model is based on the *Totally Asymmetric Exclusion Process* (TASEP, see, for example [24], [25], and subsequent studies [32]. In the TASEP, initiation time as well as the time a ribosome spends translating each codon is exponentially distributed (mean translation times are of course is codon dependent). In addition, ribosomes span over several codons and if two ribosomes are adjacent, the trailing one is delayed until the ribosome in front of it has proceeded onwards (Figure 1A, Methods, see also Text S1).

Despite its rather simple description, the mathematical tractability of the model described above is poor and full, large scale, simulations of it are relatively slow. In order to allow for analytical treatment and in order to reduce simulation times, we introduced two simplifications. First, instead of describing the dynamics at the level of a single mRNA molecule we describe the dynamics after it was averaged over many identical mRNA molecules (Methods). Second, we limit ourselves to a spatial resolution that is of the size of a single ribosome. These simplifications will be further explained and justified later.

The simplified model, entitled *Ribosome Flow Model* (*RFM*), is illustrated in Figure 1 B–C. mRNA molecules are coarse-grained into sites of 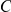 codons; (in Figure 1B *C = 3*); in practice, as we discuss with more details latter, we use *C* = 25 (unless otherwise mentioned), a value that is close to various geometrical properties of the ribosome such as its footprint on the mRNA sequence and the length of its exit channel [7], [14], [22], [33], [34], [35]. As we report later, the choice *C* = 25 is not arbitrary and was made since it gives the best predictions of protein abundance levels.

Ribosomes arrive at the first site with initiation rate 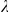 but are only able to bind if this site is not occupied by another ribosome. The initiation rate is a function of physical features such as the number of available free ribosomes [7], [36], [37], the folding energy of the 5′UTRs [6], [20], the folding energy at the beginning of the coding sequence [6], [20], [38], [39] and the base pairing potential between the 5′UTR and the ribosomal rRNA [40]. As some of these features and their combined effect are unknown and out of the scope of this paper, we assume a global initiation rate or infer the initiation rate from the coding sequences (as we show in the section ‘Optimality of the translation machinery’). We do so for the sake of simplicity and in order to avoid over-fitting of data.

A ribosome that occupies the 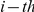 site moves, with rate 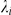, to the consecutive site provided the latter is not occupied by another ribosome. Transition rates are determined by the codon composition of each site and the tRNA pool of the organism. Briefly, taking into account the affinity between tRNA species and codons, the translation rate of a codon is proportional to the abundance of the tRNA species that recognize it (Figure 1, see more details in the Methods section).

Denoting the probability that the 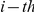 site is occupied at time 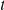 by 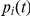, it follows that the rate of ribosome flow into/out of the system is given by: 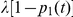 and 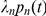 respectively. The rate of ribosome ‘flow’ from site 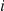 to site 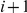 is given by: 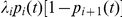 (see the Methods section). As we discuss in details (see the Methods section and Figure 1D), the *RFM* and the full TASEP model, give similar predictions, yet the *RFM* runs markedly faster.

In this paper we focus on the steady state solution of the equations presented in Figure 1C and specifically in the rate of protein production at steady state. Steady state is a widely used assumption in cases like these (see, for example, [7], [32], [33]) and is hence a good starting point for a large scale study as the one conducted here. In addition, a pioneering analysis that took into account mRNA degradation and was not based on the steady state assumption, was unable to improve the predictive power of the model with respect to existing data (Methods). We note however, that this line of investigation is far from being exhausted and that it should be revisited once degradation rates of mRNA molecules and proteins become available (this data is currently lacking for the vast majority of genomes and heterologous genes).

We denote the steady state site occupation probabilities by 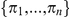 and the steady state ribosome flow through the system by 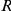. The latter denotes the number of ribosomes passing through a given site per unit time and we note that this rate is nothing but the steady state rate of protein production.

### Basic Properties of the Ribosome Flow Model

One advantage of the *RFM* is its amenability to *both* analytical and numerical analysis. In particular one can study ribosome density profiles and protein production rates from the equilibrium dynamics of the translation process. The Methods section describes how to solve the model analytically under steady state conditions; in this section we discuss some of the basic properties of the solution.

#### The behavior of the model under very low and very high initiation rates

A central debate in the field is about the rate limiting stage of gene translation: *i.e.* is it the initiation stage or the elongation stage (see, for example, [6]). Analysis of our model demonstrates that, in principle, both cases are possible.

**Figure 2:**
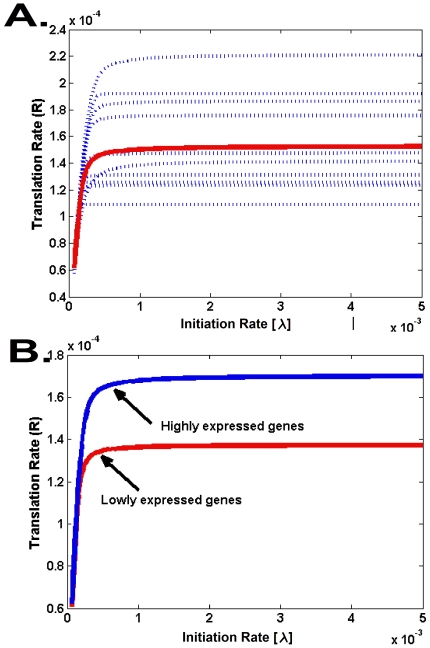
The effect of the initiation rate on the translation rate and elongation rate capacity. A. The figure depicts ten typical profiles of translation rate *vs.* the initiation rate 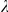 (*blue) in S. cerevisiae* genes; the mean genomic profile is shown in red. As can be seen, for very small 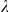 values all genes have similar translation rate (mainly determined by λ and not by the codon-bias), whereas for larger λ translation rates differ among genes and asymptotically converge to the elongation rate capacity. B. The predicted translation rate for highly (top 25%, Blue line) and lowly (lowest 25%, Red line) expressed genes.

As can be seen in Figure 2A, at very low initiation rates, 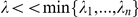, the initiation rate, 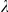, is the rate limiting step of the translation process (i.e. it is the bottleneck and the translation rate is determined by it). Thus, the translation rate is approximately given by 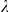
_._ On the other hand, at high initiation rates, 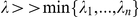, the rate limiting step is the elongation (“the flow from codon to codon”); in this case, the rate of protein translation converges to a constant that is determined by the set of elongation rates 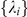 (Figure 2A; see some more technical details in the Methods section).

#### The elongation rate capacity of a coding sequence

One important feature that was discovered by implementing our model is the fact that each gene has a *different* translation elongation *capacity*. This *capacity* is the *maximal* translation rate of the gene, achievable for infinitely large 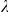. In effect, one needs not go to “infinitely large” values of 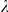 since the limiting capacity is already achieved for finite and biologically feasible values. As can be seen in Figure 2A (for large 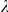), the capacity is a *finite* number that depends on the mRNA sequence; in addition, for each gene there is a possibly *different* 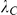, such that for every initiation rate 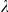 above 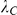, the elongation capacity is roughly equal to the maximal elongation capacity. As expected, Figure 2B shows that the elongation rate capacity of highly expressed genes is higher than the capacity of lowly expressed genes (*S. cerevisiae*; Methods).

### Predicting Translation Rates, Protein Abundance and Ribosome Densities of Endogenous Genes

#### Translation rates and protein abundance

The model was first evaluated by an analysis of three organisms for which large scale Protein Abundance (PA) measurements are available: *E. coli*, *S. pombe* and *S. cerevisiae* (Methods). It is important to note that direct measurements of translation rates are not available. However, as explained in the Methods section, the protein abundance of a gene is expected to increase monotonically with its translation rate. Thus, a good predictor of translation rate is expected to have a high *Spearman* correlation with the corresponding protein abundance. Indeed, throughout the paper we mainly report correlation of *RFM* translations rates with protein abundance (Methods). We compare the predictions of the *RFM* to the predictions of other commonly used predictors.

In each case, genes were divided into groups/bins (of equal size) according to their expression levels and the number of protein abundance measurements (a larger number of measurements, e.g. the data of *S. cerevisiae*, enables more bins); in each group the correlation between the predictions of the model and the actual protein abundance level was computed. The predictions of the *RFM* are compared with those of the *tAI*, which is the current state of the art, codon bias based, PA predictor [7], [20], [26], [27], [29], [30], [41]. The RFM and *tAI* share resemblance in the sense that they are both based on codon adaptation to the tRNA pool. However, in contrast to the RFM, the *tAI* is not sensitive to the order of codons or to the effect caused by ribosome jamming. The *tAI* is also a central component in other PA predictors that incorporate additional genomic features such as mRNA levels and evolutionary rates [26]. Thus, whenever the predictions of the *RFM* are better than those of the *tAI*, it can beneficially replace the latter as a component within a more sophisticated predictor.

**Figure 3:**
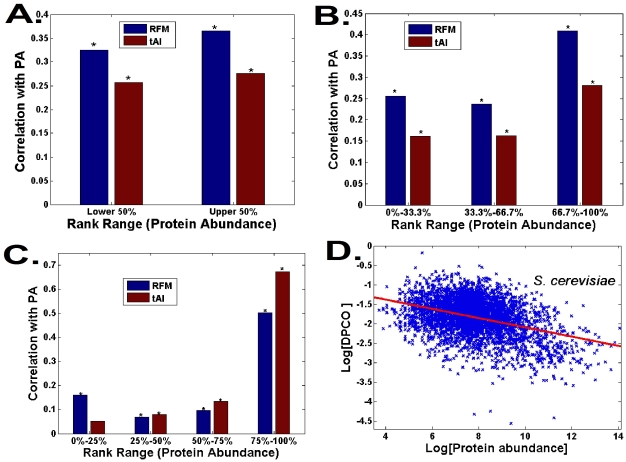
Prediction of protein abundance of endogenous genes by the *tAI* [27] and by the ribosome flow model (RFM). We compare the *RFM* to the *tAI* (insensitive to codon order), the *RFM* also outperformed other predictors, such as the Bottleneck and the Mean Speed (see definitions in the Methods section; see Figure S1). The predictions were obtained for groups of genes with different levels of protein abundance in different organisms; in each organism all bins are of equal size; organisms with a larger number of measurements enable more bins. A. Predicting protein abundance of *E. coli* endogenous genes. B. Predicting protein abundance for *S. pombe* endogenous genes C. Predicting protein abundance for *S. cerevisiae* endogenous genes [16]. D. Sensitivity to codon order *vs.* protein abundance in *S. cerevisiae*.

As can be seen in Figure 3, in the vast majority of organisms and across expression levels, the *RFM* outperforms the *tAI* (and other predictors that are based on codon bias). Specifically, in *E. coli* the global correlation between PA and the predictions of the *RFM* is R = 0.54 (p<10^−16^) *vs.* R = 0.43 (p<10^−16^) for the *tAI* (408 genes with PA data). In addition, when subdividing into expression levels, correlations are consistently higher in all subgroups (Figure 3). In *S. pombe* results were similar: the correlation with PA was higher for the RFM, R = 0.63 (p<10^−16^) *vs.* R = 0.56 (p<10^−16^) for the *tAI* (1465 genes with PA data). In addition, correlations are higher in most of the expression level subgroups (Figure 3).

In the case of *S. cerevisiae* the *tAI* performs better than the *RFM* only for the most highly expressed genes. Nevertheless, it is the *RFM* (and not the *tAI*) that yields significant correlation with protein abundance in most of the other ranges (see Figure 3C). This may be due to the tendency of highly expressed genes in *S. cerevisiae* to be more robust to permutations of the codons' order (see discussion in the next section) and due to the fact that the *tAI* was specifically tailored and optimized for *S. cerevisiae* [27].

Finally, *RFM* is seen to outperform the *tAI* also when mRNA levels are controlled for and when the product of the predicted translation rate with the mRNA level of the transcript is used as the PA predictor; see Text S2 and Figures S21, S22, S23, S24, and S25.

#### The effect of codon order on translation rates

All common measures of translation rate/translation efficiency/codon bias (see, for example, [27], [28]) predict that PA increases with the relative incidence of ‘fast’ codons along the transcript. Recently, it has been suggested that codon order (in addition to content) may regulate gene translation via the effect of ribosome jamming [7], [22], [23]. For example, slower codons at the end of the mRNA, may render the transcript prone to more ‘traffic jams’ and thus decrease the translation rate. Previous studies have attempted to estimate the effect of codon bias in the case were synonymous codons are randomly permuted and the final protein product does not change [6], [21]. Nevertheless, common measures of translation rate are not sensitive to codon order and so a direct estimation regarding the effect of the latter on the translation rate is still lacking.

In this section, we aim at isolating the effect of codon *order* on the translation rate. In other words we would like to answer the following question: is there a difference between the translation rates of two mRNA transcripts that are characterized by identical codon content but different codon order. To this end, we applied our model on random permutations of native mRNA transcripts. This was done for each gene separately, in order to compute the standard deviation in the predicted PA for the set of randomly permuted transcripts. Results are given in percentages (*i.e.* normalized by the original PA; see the exact details in the Methods section). We named this measure *DPCO* (dependence of protein abundance on codon order). We emphasize again that DPCO analysis cannot be performed using common measures of translation rate/translation efficiency since these are only sensitive to the codon content which was left unchanged by the permutation process.

A *DPCO* index of 20%, for example, means that we can quite easily get a 20% change in the gene's PA just by changing the order of its codons, and probably get a 40% change in PA by optimizing the latter with respect to codon order. Codon permutations may change the resultant protein; nevertheless, the *DPCO* gives a large scale estimation of the *distinct* effect of codon order on protein production rates and protein abundance.

Analysis of several organisms revealed that the *DPCO* of endogenous genes is surprisingly high. The mean *DPCO* is 16.35% in *E. coli* (*stdev* is 8.43%: in 10% of the genes the *DPCO* is more than 28%); the mean *DPCO* is 13.7% in *S. pombe* (*stdev* of is 4.6%: in 10% of the genes the *DPCO* is more than 19.25%); the mean *DPCO* is 17.7% in *S. cerevisiae* (*stdev* 7.92%: in 10% of the genes the *DPCO* is more than 27.46%). These results highlight the importance of incorporating codon order into models of translation rates as they support the hypothesis that one can profoundly affect the translation rate just by reordering the codons in the transcript.

In the previous section we found that the *tAI* performs well mainly for highly expressed genes; it is possible that this result is partially related to the fact that translation efficiency is less affected by codon order in these genes. We found a significant *negative* correlation (*S. cerevisiae:* r = −0.31, p<10^−16^; *E. coli*: r = −0.22, p = 9.4 10^−6^) between *DPCO* and protein abundance of genes (Figure 3D), demonstrating that in these organisms protein abundance of highly expressed genes (whose expression was predicted relatively well by the *tAI*) is less dependent on codon order than it is in lowly expressed genes. Thus, the result reported in this section support the usage of models such as the *RFM* for predicting the translation rate of endogenous genes that are *lowly* expressed (see also Text S3 and Figures S26 and S27).

It is important to note that the predictions reported in this section should be confronted with experimental measurement when these become available. However, in light of the fact that controlled design of ‘wet experiments’, that would allow the validation of the predictions presented above, is far from being trivial (*e.g.* changing the order of codons may influence other features of the coding sequence), the estimations reported here are particularly interesting.

#### Coarse graining and genomic ribosomal density profiles

**Figure 4:**
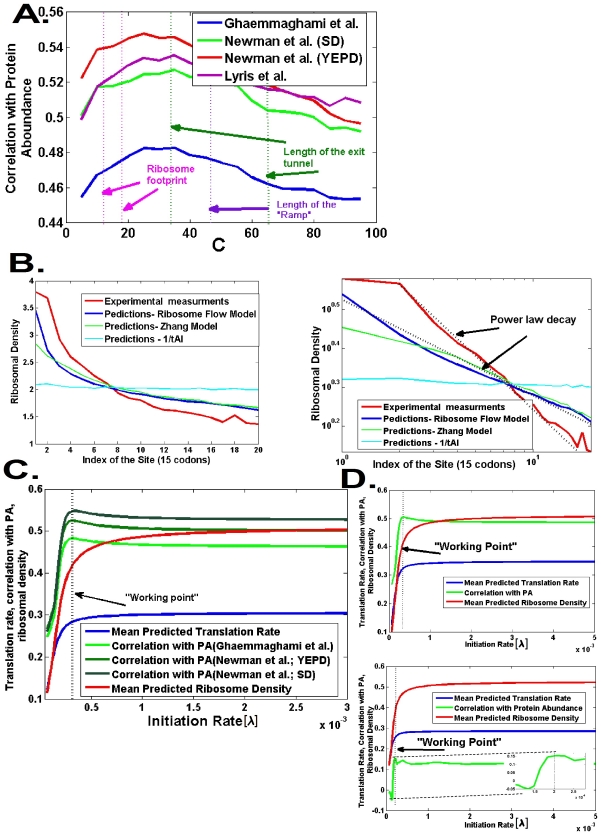
Relations between various quantities predicted by the *RFM* and biological measurements. A. Correlation between protein abundance [16] [15] [66] and the translation rate for various values of the coarse graining parameter (*C* in Figure 1); the best results are observed for values which are similar to various geometrical properties of the ribosome (the dashed lines in the figure). B. Right: The *RFM* predicts the genomic ribosomal density profile [14] better than the *tAI* or the model of Zhang *et al.* [33]; all were normalized to have the same mean. Left – the 5′ region of the genomic ribosomal density profile and the predicted genomic profile of the *RFM* appear linear on a log-log scale. We used a site size of 15 codons (similar to the size of the ribosome) and a 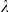 (initiation rate) value that was independently found to optimize the correlation with protein abundance. C. The relation between λ (associated with the number of available ribosomes in the cell), genomic mean of the translation rate, and the genomic mean of the ribosomal density. D. Initiation rate (λ), translation rate, and ribosomal density for highly expressed genes (up) and lowly expressed genes (down).

Figure 4A depicts the correlation between translation rate predictions of our model and protein abundance in *S. cerevisiae* for different values of the coarse graining parameter C (*C* in Figure 1). Interestingly, the optimal correlation is obtained for sites of size 25–35 codons (and is supported by jackknifing test; Methods). This value is similar to length scales associated with the ribosome such as its footprint on the mRNA sequence [7], [14], [33], [34], [35] (between 11 and 18 codons), the number of amino acids associated with the exit channel of the ribosome and its length [42], [43], [44], [45] (between 30 and 71 codons), and the length of the ‘ramp’ at the beginning of genes corresponding to the optimization of ribosome allocation [7], [44] (around 50 codons); similar results were obtained for other organisms as well Figures S2, S3. This result provides further support for the validity of our model. Specifically, this result is consistent with the assumption that site size in our model should be of the same order of magnitude as the ribosome size since physically this is the relevant length scale in the analyzed biological system.

In the next step, we studied how well the *RFM* predicts the shape of the genomic profiles of ribosome density. To this end, predictions of our model and other models were compared to a genomic ribosomal density profile that was generated based on, single nucleotide resolution, large scale measurements of ribosomal density (Methods, [14]; Figure 4B).

Strikingly, as depicted in Figure 4B, although all models predict that there is a decrease in ribosome density from the 5′ end to the 3′ end of the mRNA transcript, the gap between the real profile of ribosomal density and the profile predicted by the *RFM* is significantly smaller than the one obtained by Zhang's model ([33] 0.26 *vs.* 0.3; Wilcoxon test p-value<0.0001) or from the graph corresponding to per-codon mean genomic 1/*tAI* [7] (0.26 *vs.* 0.54; Wilcoxon p-value<0.0001). Specifically, it seems that both the genomic ribosome density profile and the *RFM* predictions are characterized by a non exponential decay from the 5′ end of the coding sequence to the 3′ end of the coding sequence and are seen linear on a log-log graph (Figure 4B; see also Text S4). In contrast, the *tAI* predicts a much slower mean genomic decrease rate (Figure 4B). This result further supports the *RFM* as a model that describes the physics of gene translation better than previously suggested models (similar results were obtained for ribosome density profiles obtained under starvation conditions; see Figure S4).

#### Optimality of the translation machinery

One basic translation-related feature of a gene is the mean, steady state, ribosome density on the transcript. This value can be predicted by 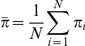 (the mean probability that a site will be occupied by a ribosome). In the RFM, 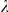 models the effect of the number of free ribosomes on the initiation rate. Given that there are more ribosomes, the initiation rate would increase since the rate in which ribosomes arrive at the 5′ end of the mRNA is proportional to the number of free ribosomes. What are the relations between 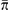, 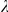, and the translation rate in general? And in particular, what is the actual ‘working point’ (in the 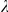,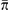,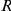 parameter space) of the translational machinery?

Figure 4C depicts the translation efficiency at different values of 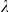. At low 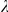 levels the translation rate and ribosome occupancy increase monotonically with 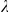. However, as was demonstrated before [46], after a certain point the system reaches saturation – increasing 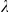 does not result in a further increase of the translation rate or the mean genomic ribosomal density.

Interestingly, the correlation between the predicted translation rate and the measured protein abundance of yeast is maximal exactly before the onset of saturation (Figure 4C). This fact may suggest that the translation machinery is tuned to work in the vicinity of this point. Thus, this may indicate that there is *global optimality* of the initiation rate in *S. cerevisiae* (similar results were obtained for other organism: *S. pombe*, *E. coli*, *Human* liver; see Figures S5, S6, S7).

We note that the pre-saturation point is optimal from an engineer's point of view. The basic reasoning for this follows from the fact that going below the pre-saturation dramatically decreases the rate of protein production. On the other hand, going above and beyond the pre-saturation point, would require additional resources from the cell. This investment however, will have no effect on the mean protein production capacity and will therefore be in vein.

For a *given* initiation rate, 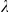, faster codons (*i.e.* higher 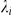 or higher *tAI*) should decrease the ribosomal density due to the reciprocal relation between translation rate and ribosomal density [7], [20]. Thus, *under the assumption of a global initiation rate*, and since highly expressed genes have more efficient codons, we expect a *negative* correlation between expression levels of genes and their ribosomal density. However, in practice this is not the case - the correlation between translation efficiency (*tAI*) and ribosomal density is positive and significant (for example, r = 0.46; p<10^−16^ for the ribosomal density measurements of [10] and the mRNA measurements of [47]). This result suggests that the initiation rate (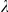) of highly expressed genes is *higher* than that of lowly expressed genes. Refining our analysis, we will now revisit, and relax, the simplifying global initiation rate assumption we have made so far.

Given a set of genes (*e.g.* highly expressed genes) the estimated initiation rate 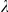 of this group is the one that gives the best correlation between the predicted translation rates and protein abundance. We estimated the initiation rate in highly expressed genes (top 20%) and in lowly expressed genes (lowest 20%; Figure 4D). Indeed the predicted initiation rate of the highly expressed genes is higher than that of the lowly expressed genes (0.00035 *vs.* 0.0002) while the resulting predicted ribosome density is also higher for the highly expressed genes (0.42 *vs.* 0.36). Thus, in practice (at the ‘working point’), our model predicts that highly expressed genes, that are equipped by faster codons and thus characterized by higher translation rates, are also characterized by higher ribosomal densities as their initiation rate is higher. The fact that in highly expressed genes ribosomal densities are higher, suggests that in these genes, elongation rate is more rate-limiting (relatively to lowly expressed genes). This result explains why in highly expressed genes codon bias should be a better predictor of translation rate (as was shown in Figure 3).

As shown in 3.2, different mRNA transcripts are characterized by different *translation elongation capacities*. Here, based on the correlation between translation rates and protein abundance, we have just shown that, on average, the predicted 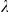 is the one for which this capacity is almost fully achieved (*i.e.* 93% of the capacity is attained in *S. cerevisiae*). This rule enables inference of the initiation rates of *individual* genes: *e.g.* in *S. cerevisiae*, the predicted initiation rate of a gene is the one for which 93% of its elongation capacity is attained (in other organisms the rule is similar; Methods).

Strikingly, the predicted initiation rate of genes significantly correlates with their protein abundance (*S. cerevisiae* r = 0.29, p<10^−16^; *S. pombe* r = 0.41, p<10^−16^; *E. coli*, r = 0.34, p = 8 * 10^−13^ Figure S8, S9, S10); *i.e.* highly expressed genes have higher initiation rates. In addition, the predicted initiation rate correlates with the predicted ribosomal density (*S. cerevisiae* r = 0.72, p<10^−16^; *S. pombe* r = 0.6531, p<10^−16^; *E. coli*, r = 0.3379, p<10^−16^, Figure S11, S12, S13, Methods) – i.e. highly expressed genes are characterized by higher ribosomal density (the correlation between predicted ribosome density and protein abundance of genes: *S. cerevisiae* r = 0.19, p<10^−16^; *S. pombe* r = 0.104, p = 2.44*10^−4^; *E. coli*, r = 0.32, p = 2.1 * 10^−11^; Figure S14, S15, S16). These results demonstrate again that the predictions of our model are in accord with the experimental observation that highly expressed genes have higher initiation rate and higher ribosomal density (mentioned above) [10].

#### Analysis of heterologous gene expression

As was demonstrated in section ‘Predicting translation rates, protein abundance and ribosome densities of endogenous genes’, the *RFM* is considerably better (than current state of the art predictors) at predicting the PA of lowly expressed genes with coding sequences that differ from the optimal design. This is usually the case when a gene from one organism (*e.g.* Human) is expressed in a different organism (*e.g. E. coli*; see for example, [5], [6], [21], [48]), a procedure known as heterologous gene expression. Heterologous gene expression allows the use of mRNA ‘libraries’ that are composed of different variants of the *same heterologous gene*. In this method of expression, control for various properties is already ‘built in’. In particular, the amino acids composition of the translated protein remains unchanged.

In this section, we use our model to analyzing two cases of heterologous gene expression, demonstrating that the *RFM* markedly outperforms the *tAI* (and other alternative predictors). In what follows, we emphasize the differences between endogenous and heterologous genes. As we demonstrate, the gap between the predictions of our model and those of the tAI is higher for heterologous genes. This property of the RFM, demonstrates the potential biotechnological applications of our approach - predicting the protein abundance of heterologous gene expression.

**Figure 5:**
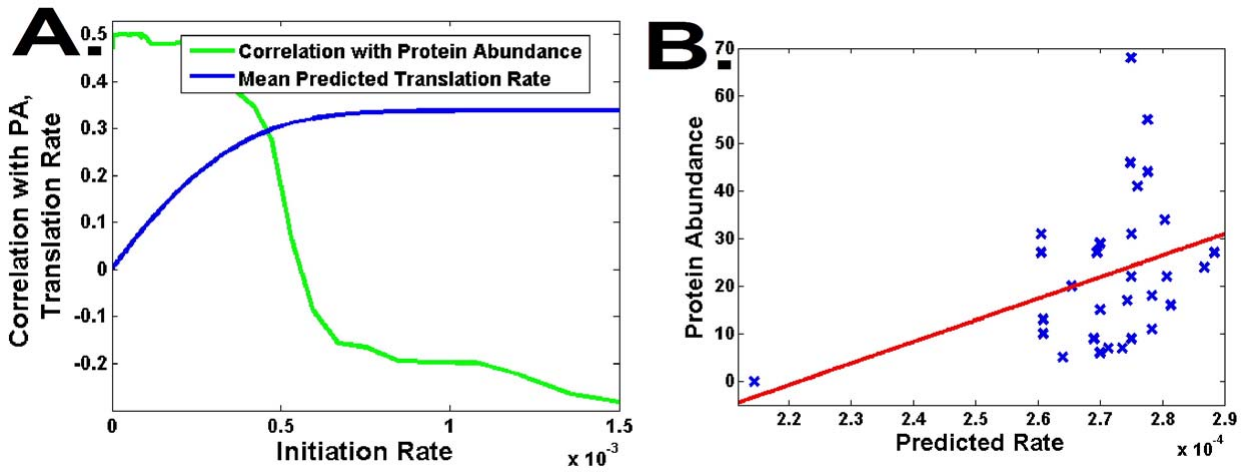
Analysis of the data of Welch *et al.* [**21**] by the *RFM* model. A. The translation rate and the correlation with protein abundance as a function of 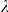. B. A dot plot - predictions of the *RFM vs.* protein abundance.

We analyzed the data of Welch *et al.* [21], a large library of genes encoding DNA polymerase of *Bacillus* phage pi29 proteins, results are shown in Figure 5. All the genes encode the same amino acid sequence but each of them has a different codon composition. Although it was reported that there is *no correlation* between codon-bias or folding energy and protein abundance in this dataset [20], [21], we found a significant correlation between the predictions of the *RFM* and protein abundance (r = 0.5, p = 0.004).

Correlation is significant only for very low initiation rates, suggesting that initiation (or other variable, as was suggested in [21]) is rate limiting in the translation of these genes. In contrast to what was observed for *endogenous genes* (Figure 4), the point with maximal correlation between the prediction of the model and PA is *not* the pre-saturation point. This result demonstrates that the coupling between translation rate and initiation rate is an evolutionarily *selected* trait, and is hence not observed in *heterologous* coding sequences.

We continued with an analysis of the data by Burgess-Brown *et al.* [48], who optimized the codons of 31 human genes in order to express them in *E. coli* [48]. In this study, the protein abundance of 18 genes improved, that of one gene *decreased*, and the other 12 did not change in a detectable way. The Spearman correlation between the direction of the change in *PA* and the predicted fold change (*i.e.* the ratio between the translation rate before and after the optimization) of the *RFM* was 0.45 (empirical p-value = 0.019) while the correlation with the fold change according to the *tAI* was only 0.34 (empirical p-value = 0.077; Methods). This result demonstrates once more that the *RFM* is a particularly useful tool for the analysis of heterologous gene expression (see also Text S5).

#### Condition-specific translation rates in S. cerevisiae

When the yeast *S. cerevisiae* is grown on glucose-based media, it first utilizes the available glucose, growing by fermentation. When most of the glucose has been consumed it undergoes a metabolic change, called diauxic shift, in which its metabolism shifts to respiration. This is accompanied by wide changes in gene expression and tRNA abundance [7], [49]. In [7] we focused on the similarities between the tRNA pools in different stages of the diauxic shift (for example, the Spearman correlation between the tRNA abundance at time 0 and the tRNA abundance after 9 hours is 0.9, p-value 6*10^−15^ ; *i.e.* 0.81 of the variance in the tRNA pool at time 9 hours can be explained by the tRNA pool at time 0 hours). In the current study we analyze the *dissimilarities* between the tRNA pools during different stages of the diauxic shift. Changes in the tRNA pool due to the diauxic shift lead to changes in the translation rate of different codons. The total effect of these changes is related, among other factors, to the order of codons along the mRNA transcript and therefore cannot be inferred completely by the *tAI*.

Here, we use our model to analyze the dynamics of genomic translation rates during the diauxic shift in *S. cerevisiae* (using data from [7]). In each stage of the diauxic shift, we computed the expected translation time (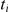) of each codon based on the available tRNA pool at that stage [7]. These times where then used in conjugation with the RFM in order to compute the mean genomic translation rate and ribosomal densities for different values of the initiation rate 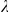.

**Figure 6:**
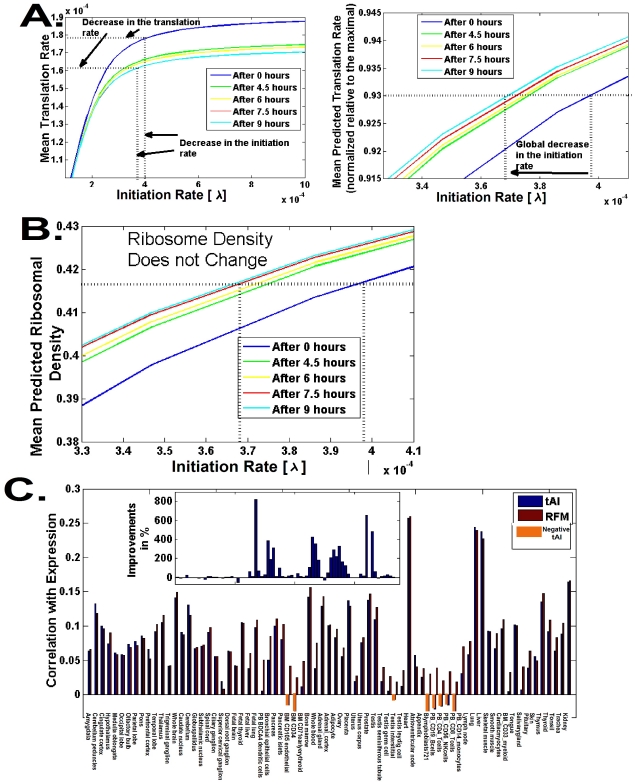
Translation rate and ribosome density during the diauxic shift in *S. cerevisiae*. A. The mean genomic translation rate as a function of the initiation rate (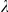) for five time points; the dotted lines correspond to the working point just before saturation (93% of the maximal production rate, mentioned in sub-section 4). B. The mean genomic ribosomal density as a function of the initiation rate (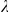) for five time points. The dotted lines correspond to the initiation rates at the working points. C. The correlation between the mRNA levels of genes in different human tissues *vs.* (a) the *RFM* predictions and (b) the *tAI* predictions. Inset: the improvement in correlation in % when using the *RFM* instead of the *tAI*.

As the new growth conditions are less optimal for the yeast we expect a global reduction in the rate of translation. The mean genomic profile of the translation rate and ribosomal density of all *S. cerevisiae* genes at five time points (0, 4.5, 6, 7.5 and 9 hours after the beginning of the experiment) during the diauxic shift, is presented in Figure 6A–B. As can be seen, all these profiles are similar to the ones reported earlier - displaying saturation of the translation rate and the ribosomal density for large 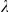.

As expected, both the predicted translation rate and the predicted number of available free ribosomes (or equivalently the initiation rate 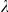) decrease during this process (Figure 6A). Interestingly, although the mean codon efficiency remains essentially unchanged during the process (a minor decreased of 0.16% in the mean genomic expected time for translating a codon), the mean production rate does *decrease*s due to changes in the initiation rate (number of free ribosomes; see details in Figure 6A) and effects related to the flow of ribosomes and the *order* of codons. In contrast, the mean predicted ribosomal density *does not* decrease as 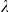 decreases (see details in Figure 6B). Thus, while the total effect under these conditions is also related to changes in mRNA levels, initiation/elongation factors and more (see [49]), our model predicts that part of the global response can be attributed to changes in the composition of the tRNA pool. Such an analysis cannot be performed by simple measures such as *tAI*.

In the next step, we checked how well the predicted *change* in translation rate of genes during the Diauxic shift correlates with the change in their mRNA levels. We compared the *change* in the predicted translation rate of genes whose mRNA levels exhibited extreme fold change (fold changes >1.8 and <1/1.8) and found that the ranked fold changes of the translation rate of the genes in these groups was also significantly different (mean fold change 1.035 *vs.* mean fold change 0.9991; p = 2.47*10^−5^). Ranking the changes in the *tAI* led to opposite result – a decrease in the translation rate of genes whose mRNA level increased and vice versa (mean fold change 0.9923 *vs.* mean fold change 1.0103), demonstrating again the superiority of our model. This result demonstrates that (1) in *S. cerevisiae*, condition-specific changes in the translation rate of genes are in accordance with the changes in their transcription levels; and (2) the *RFM*, by considering refined features such as the order of codons and initiation rates is specifically sensitive to the adaptation of an organism to a dynamically changing environment.

#### Translation efficiency in human

Finally, comparison of the predictions of the *RFM* to tissue specific mRNA levels (that are known to correlate with protein abundance and ribosomal densities [10], [14], [26]) in human demonstrated that it outperforms the *tAI* in this organism as well (Figure 6C, Text S6). Specifically, the gap between the *RFM* and the *tAI* is particularly large in germ line and immune cell types. Thus, specifically in these tissues, the *RFM* should be helpful in analyzing mutations (see, for example [41]) or SNPs (see, for example, [2], [50], [51]) that cause diseases due to problems in gene translation.

In addition, we computed the correlation between the prediction of the RFM and *protein abundance* in Human cell lines for which PA data exists [52]. The correlation between the predictions of the *RFM* and protein abundance was 0.47 (p-value<10^−16^) *vs.* a correlation of only 0.28 (p-value<10^−16^) between the *tAI* and protein abundance.

## Discussion

We described a novel analysis of large scale genomic data by a predictor/model that is based on the physical and dynamical nature of gene translation. Given the copy numbers of the tRNA genes in the host genome, our model, the *RFM*, is based *only* on codon-bias; It can hence be applied when *only* the coding sequence of a gene is available and without additional data or information. Despite its relative simplicity, we show that our model predicts features such as protein abundance in endogenous and heterologous genes better than alternative (‘non-physical’) approaches. We demonstrate that the gap between the performance of the *RFM* and alternative predictors is especially large in the case of heterologous genes; thus, it should be very helpful in the common challenge of predicting the protein abundance of potential heterologous proteins before expressing them in the desired host (see, for example, [5], [6], [7], [21], [53], [54], [55], [56]). In addition, we have demonstrated that our approach can be used for accurately inferring various variables that *cannot be inferred by the common predictors used nowadays*.

From a Systems Biology point of view, by using our model we were able to demonstrate the global optimality of the process of gene translation [6], [7], [20]. We discovered that increasing the number of available ribosomes (or the initiation rate, 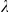) increases the genomic translation rate and the mean ribosomal density only up to a certain point. After this point, the system is ‘saturated’: adding more ribosomes/increasing the initiation rate does not result in an increase of these two variables. Quite strikingly, in all the organisms we have analyzed, the global initiation rate is optimized to the pre-saturation point. The fact that similar results were *not* observed in artificial genes supports the conclusion that this feature is under selection.

Optimality of the translation machinery is perhaps not so surprising. Protein production is a central and complex process in the cell. For example, at any given time point there are around 60,000 mRNA molecules in *S. cerevisiae* [36] that are translated by 187,000 (±56,000) ribosomes [37]. The process of gene translation consumes a very large amount of energy and thus the problem of fine tuning the number of ribosomes and the translation rate should have a significant influence on the fitness of the organisms [6], [7], [20]. Specifically, increasing the translation rate of highly expressed genes (the ‘supply’) while decreasing the number of working ribosomes/ribosomal density (the ‘cost’) should improve the fitness of an organism. It was already suggested that there is selection for improving translation efficiency of highly expressed genes relatively to lowly expressed genes (see, for example, [6], [20]). By using our model, we can actually estimate the translation cost of highly and lowly expressed genes as the ratio between the translation rate and the average number of ribosomes working on the transcript. The number of proteins produced per unit time, per ribosome, for highly expressed genes (top 20%) is 0.000162/0.42 = 0.000386 (in arbitrary units). This number is 10% higher than that of the lowly expressed genes (lower 20%; 0.000125/0.36 = 0.000347). Again, this result demonstrates ‘optimality’: as highly expressed genes produce more mRNA molecules, decreasing the cost of translation should result in a much larger effect on the fitness of the organism.

Finally, the goal of this study was to model the process of translation elongation, emphasizing the effect of codon order. In the future, in order to decrease the gap between the predictions of our models and measurements of protein abundance, we intend to develop a more comprehensive model of this process. While promising strides in this direction were already made [57], [58], may features of the translation process are yet to be accounted for. Unfortunately, large-scale biological measurements of *translation rates*, *initiation rates*, *tRNA levels*, *mRNA/protein degradation rates* and many other quantities that are related to the process of gene translation are currently unavailable. Large scale measurements that are available (*e.g.* protein abundance) are related to the modeled process (Methods), but are *indirect*. This fact hinders the implementation and validation (as opposed to formulation) of more sophisticated models. In addition, it is important to note that the ability to predict measurements of protein abundance may also be hindered due to bias and noise in the current pool of existing data (see, for example, [17], [59]). As new data accumulates, the implementation of more comprehensive models will become possible and our understanding of the translation process will deepen further.

## Methods

### The TASEP Model for Translation Elongation

In the TASEP an mRNA transcript with 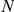 codons is modeled as a chain of sites, each of which is labeled by the index 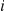, where 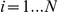. The first and last codons, 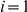, 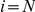, are associated with the start and stop codons, respectively. At any time, *t*, attached to the mRNA are *M(t)* ribosomes. Being a large complex of molecules, each ribosome will cover 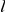 codons. A codon may be covered by no more than a single ribosome. To locate a ribosome, we arbitrarily assume that the codon being translated is the one in the ‘middle’ of the ribosome. For example, if the first, *(l+1)/2* codons are not covered, a ribosome can bind to the first codon on the mRNA strand, and then it is said to be “on codon 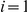
”. A complete specification of the configuration of the mRNA strand is given by the *codon occupation* numbers: 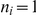 if codon 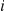 is being translated and 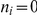 otherwise. Note that when 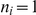 the *(l−1)/2* codons before and after codon 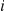 are covered by the ribosome that is on site 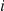. Since these codons are not the ones being translated, the codon occupations numbers for them are equal to zero.

We will now specify the dynamics of the TASEP model. A free ribosome will attach to codon 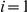 with rate 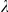, provided that the first 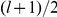 codons on the mRNA are empty. An attached ribosome located at codon 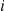 will move to the next codon 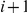 with rate 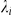, provided codon 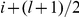 is not covered by another ribosome. In case 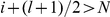 (ribosome is bulging out of the mRNA strand) an attached ribosome will move to the next codon with rate 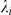.

In order to simulate this dynamics, we assume that the time between initiation attempts is distributed exponentially with rate 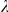. Similarly the time between jump attempts from site 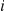 to site 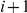 is assumed to be exponentially distributed with rate 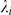 (The exponential distribution is of course, an approximation as the process of translating a single codon involves more than one step [1]). Note that in the case of 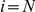 the jump attempt is in fact a termination step. We define an “event” as an initiation, jump attempt, or termination step. From our definition it follows that the time between events is exponentially distributed (minimum of exponentially distributed random variables) with rate 
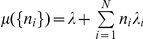
. Note that a jump attempt from codon 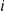 can only be made if there is a ribosome translating this codon and hence the rate 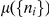 depends on the set of *site occupation* numbers.

The probability that a specific event was an initiation attempt is given by: 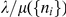. Similarly, the probability that a specific event was a jump attempt (or termination event) from site 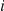 to site 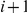 is given by 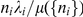.

At each step of the simulation, we determine the nature of the event and the time passed till its occurrence by these rules. The set of *site occupation* numbers are then updated accordingly and the simulation proceeds to the next event. For example if an initiation attempt was made, we check if the first 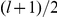 codons on the mRNA are not covered. If so, we set 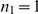, otherwise the attempt fails and 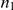 remains as is. If a jump attempt from codon 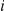 to codon 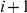 was made, we check if site 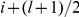 is not covered. If so, we set 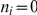 and 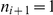, otherwise the attempt fails and 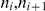 remain as is.

Starting with an empty mRNA strand we simulate the system for 250,000 steps (events). The system is then simulated for an additional 1,000,000 steps where we keep track of the total number of terminations and the total time that have passed from the point this phase have started. The steady state rate of protein production was determined by dividing the number of termination events by the total time that has passed. The number of steps in the first and second stages was determined after observing that increasing the number of steps fourfold had a negligible effect on the predicted protein production rate.

### The Ribosome Flow Model

#### Physical interpretation of the ribosome flow model

Assume that a ribosome is *C* condos long and that the mRNA strand is positioned such that translation takes place from left to right. The ribosome flow model assumes that a ribosome lands on the mRNA strand such that the first codon is located at the middle of the ribosome. The ribosome now needs to translate *C* codons in order to have its middle point reach codon *C+1*. This way the right edge of a newly arriving ribosome can be positioned next to the left edge of the ribosome who has just translated the first C codons. We now coarse grain the mRNA strand into two groups of sites (‘chucks’):

- 1…(C+1)/2,1+(C+1)/2…C+(C+1)/2,1+C+(C+1)/2…2C+(C+1)/2,…
- 1…C,C+1…2C,2C+1…3C,…

The flow of ribosomes from site 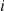 to site 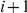 in the group A is determined by:

1. The occupation probabilities of these sites. The higher the occupation probability of site 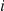 (more attempts per unit time to flow from site 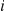 to site 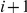) the higher the flow to site 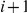. The higher the occupation probability of site 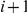 (more chances that a ribosome will be blocked by another ribosome residing in site 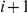 when attempting to flow from site 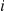 to site 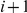) the lower the flow emanating from site 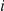.
2. The translation time of the *C* codons that belong to 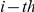 site in group B, the lower the time the higher the flow.

These ideas are expressed quantitatively by equation (1): 
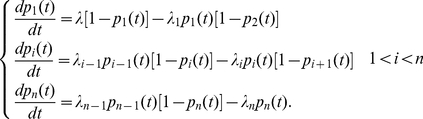
(1)


#### Analytic solution of the ribosome flow model

In order to proceed we recall that in steady state the occupation probabilities are constant in time and equal to 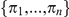. Denoting the steady state rate of protein production by 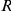 it follows that:
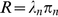
(2)This rate is also equal to the steady state rate at which ribosomes leave the mRNA strand (after translating the entire sequence). At steady state the left hand side of equation (1) vanishes and we get:
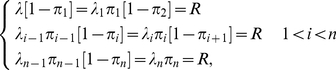
(3)where we have also used equation (2). An interesting conclusion follows from equation (3), since for every site 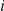: 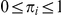 (probability is always non-negative and not larger than one) the steady state rate of protein production is limited by slowest rate in the system:
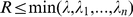
(4)Solving equation (3) for 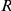 we obtain:
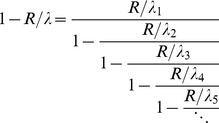
(5)Equation (5) is the starting point for the analytical analysis of the model as is further described below. Note that in principle equation (5) can be solved numerically for 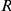 given the set 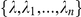, the unknown steady state occupation probabilities 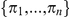 can then be computed via equation (3). In practice however, we have numerically solved the original set of differential equations (equation (1); Figure 1C).

#### Solving equation (1) numerically

In order to obtain the set of steady state occupation probabilities, 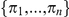, and the steady state rate of protein production, 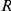, we solve equation (1) numerically using Matlab. Equation (1) is treated as an ordinary differential equation for the vector 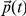 whose entries are the occupation probabilities: 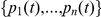. We start from an mRNA strand which is empty of ribosomes, 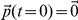. The occupation probabilities are then found for a set of later times using equation (1) and Matlab's ordinary differential equation solver. The process stops when the vector 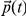 converges to the vector of steady state occupation probabilities. More accurately, we stop the process for a time 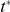 for which 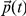 is constant (up to some prefixed numeric error threshold) for every 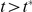. The vector of steady state occupation probabilities and the protein production rate are then taken as: 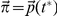 and 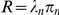.

#### Analytical analysis of low and high initiation rates

An interesting question goes to the behavior of the model in the limits of low/high external ribosome flux. The limit of low ribosome flux is mathematically given by: 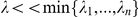. In this limit the rate of protein production may be approximated by *R*≈λ and it is hence insensitive to codon bias. In other words, the genomic rate of translation is equal to the rate of ribosome arrival since this is the latter is the rate limiting step of the process. In order to derive this result we first note that in this limit 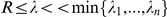 by use of equation (4). It follows that 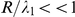 and we may hence approximate by neglecting the right hand side of equation (5). The requested result then follows as is further illustrated in Figure 2A.

The limit of high ribosome flux is mathematically given by: 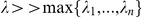
_._ In this limit the rate of protein production converges to a transcript specific constant 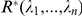 that does not depend on the ribosome flux 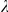 (Figure 2A). Under these circumstances the rate of protein production is strongly affected by codon composition and codon arrangement along the mRNA molecule. In addition, the independence of 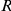 on 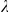 implies that above a certain threshold any attempt to increase 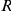 by increasing 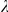 is futile. Since increasing 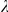 comes with the cost of spending valuable resources on maintain a large ribosome pool cost/benefit considerations will set a clear physiological upper bound on 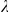 (see also section ‘Optimality of the translation machinery’). In order to understand the behavior of the protein production rate in this limit we first note that 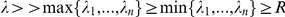 by use of equation (4). It follows that 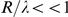 and we may hence approximate by neglecting this term in the left hand side of equation (5). We now see that 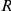 is a solution to an equation that does not contain the ribosome flux 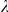 as was argued above. This result is further illustrated in Figure 2.

### The TASEP Model vs. the RFM

The TASEP model mentioned above is a generalization (elongated particles and site dependent rates) of the simple TASEP model (see, for example, [60]). In the case of the ribosome flow model, we make two approximations. The first is coarse graining (dividing into chunks/sites), this approximation is quite common and was applied to various physical and biophysical problems. The second approximation is nothing but the mean field approximation. This means that in order to write the master equation for our model (Figure 1C) we have implicitly neglected the fact that there could be *correlations* between sites. We hence write approximate equations for the average (over many identical mRNA systems) occupation probabilities. Doing so, we assume that the probability that site *i* is occupied/empty and that site *i+1* is occupied/empty is well approximated by the probability that site *i* is occupied/empty times the probability that site *i+1* is occupied/empty. Although in general this is not always true, this approximation is also common in the TASEP literature.

### RFM with Abortions

Within the framework of the RFM, abortions were modeled by adding an abortion probability to the model. The abortion probability determines the percent of ribosome-ribosome collisions that will result in abortion, i.e., in premature detachment of the ribosome from the mRNA strand. Mathematically, abortion adds the following term to the model: 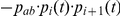 where 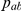 is the abortion probability. For every 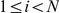 this term is added to the *i*-th and (*i+1*)-th rows of equation (1). This modification of the RFM corresponds to mutual abortion, *i.e.* for a situation where after an abortive collision both ribosomes will stop processing the mRNA transcript. Scanning different values for 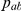, we discovered that maximal correlations were obtain in the case of 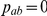, i.e. in the limit were abortions due to ribosome-ribosome collisions are negligible.

### mRNA Half Life – Steady State Revisited

In order to examine the steady state assumption (within the limitations of existing data), we analyzed the *RFM* model without it. Analysis was performed on the *S. cerevisiae* data where we simulated the model only for a time period proportional to the half life of the corresponding transcript [61]. In this case, steady state was not achieved and the translation rate was taken as the mean translation rate over the elapsing time period. This modification however, was unable to improve the predictive power of the model and in effect resulted in an opposite outcome.

### Zhang Model

Zhang model [33] similar to the TASEP model with the only change that the codon translation times are deterministic.

### The Relation between Translation Rate and Protein Abundance

Here we would like to discuss the relation between translation rates and protein concentration/abundance. In what follows we will provide justification for the intuitive expectation that protein abundance should stand in high positive correlation with translation rates. Generally speaking, protein abundance levels are determined by a balance between protein production and degradation rates. Fixing the degradation rate, protein abundance levels will rise when the production rate is increased. Fixing the production rate, protein abundance levels will decrease when the degradation rate is increased. This said, one must also bear in mind that protein degradation rates are unavailable in most of the analyzed cases. And so, any current real data analysis is forced to average out the effect of protein degradation and focus on the contribution of the production rate to the determination of protein abundance levels.

Let 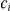 denote the concentration of protein 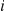 and let us assume that this protein is translated from a certain mRNA transcript whose copy numbers are denoted by 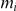. In general, the dynamics of this process may be described by the following differential equation: 
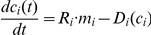
. Here 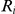 and 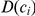 are the translation rate per mRNA molecule and the degradation rate of protein 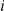 correspondingly. One possible choice for 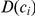 is: 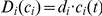 where 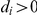 is constant. Although this is a common first order approximation we will not base our conclusions on this particular choice and would only require that 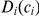 is a monotonically increasing function of the concentration 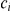. In general, the function 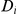 depends on the protein 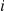, *i.e.* it can be different from protein to protein. Here however, we will replace the protein specific function 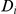 with a genomic average degradation function 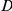 which will be assumed monotonically increasing. Note that by definition, this function does not depend on the index 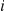.

The steady state solution of the above differential equation (with 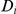 replaced by 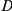) is: 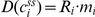 where 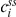 is the steady state concentration of the protein 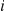. From the monotonicity of 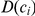 it follows that 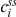 is a monotonically increasing function of 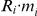.. This fact provides justification for the use of 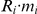 as a predictor for 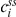, *i.e.* one expects 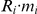 and 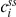 to be positively correlated. Indeed, we have shown that this predictor performs very well, see Text S2. We will now show that 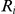 itself can also be used as a predictor for 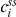, the advantage of this predictor is that it is solely based on the *coding sequence* and no additional information is required for its computation.

The set of mRNA copy numbers 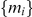 may generally depend on the set of translation rates 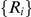, for example via the concentration of proteins that are involved in mRNA transcription and regulation. Fortunately, it is known that in endogenous genes translation rates are positively correlated with mRNA levels. Highly expressed genes are under selection to have higher mRNA levels, higher translation rate and higher protein abundance (note that this is not a causal relation; see, for example, [6]). Since mRNA levels are *positively* correlated with translation rates, higher values of 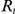 do indeed imply higher values of 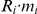 and vice versa. Since in hetrogenouse gene expression mRNA copy numbers are usually independent of the mRNA variant of the protein, a similar trend is observed in this case as well. In building a predictor which is solely based *on coding sequences*, these empirical observation provide justification for using 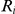 as a predictor for 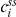. Indeed, as we have demonstrated throughout the paper, this predictor out performs other commonly used predictors.

### Data

#### Protein abundance

Protein abundance of *S. cerevisiae* was downloaded from [15], [16]; protein abundance of different versions (with different codon bias) of GFP library in *E. coli* were downloaded from [6]; Protein abundance of *S. pombe* were downloaded from [62] and the Protein abundance *E. coli* were downloaded from [17].

#### Profiles of Ribosme density

In *S. cerevisiae* were downloaded from [14].

#### Folding energies

Of the *E. coli* GFP library was downloaded from [6].

#### tRNA copy number

Of *E. coli*, *S. cerevisiae*, and *S. pombe* were downloaded from [20].

#### tRNA levels in diauxic shift

In *S. cerevisiae* were downloaded from [7].

#### Coding sequences

Coding sequences of *S. cerevisiae*, *E. coli*, and *S. pombe* were downloaded from [20].

#### Tissue specific gene expression and *tAI* in Human

The gene expression was downloaded from [63]; the corresponding *tAI* were downloaded from [30]. Inferred tissue specific tRNA pool in human liver (the tissue where the correlation between the expression levels and translation rate is the highest) was downloaded from [7], [30] based on [64].

#### mRNA levels

mRNA levels of *E. coli* were downloaded from [17]; mRNA levels of *S. cerevisiae* were downloaded from [47]; mRNA levels of *S. pombe* were downloaded from [62].

### Estimating the *tAI* Based Values That Were Used by the Model

Our measure was based on the *tAI* [27]; as describe below, we adjusted it to our model:

Let *n_i_* be the number of tRNA isoacceptors recognizing codon *i*. Let *tCGNij* be the copy number of the *j*th tRNA that recognizes the *i*th codon, and let *S_ij_* be the selective constraint on the efficiency of the codon-anticodon coupling. We define the *absolute adaptiveness*, *W_i_*, for each codon *i* as:
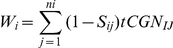
The *S_ij_*-values can be organized in a vector (S-vector) as described in [27]; each component in this vector is related to one wobble nucleoside-nucleoside paring: I∶U, G∶U, G∶C, I∶C, U∶A, I∶A, etc.

Sensitivity analysis of the *tAI* of codons to *S_ij_* -values in *S. cerevisiae* showed that one codon (CGA) is extremely sensitive to these s-values. Increasing/decreasing the s-values by +−0.5 resulted in a change of up to one order of magnitude (usually much less) in all other codons. In the case of CGA, the change was up to 4000 times higher.

The *tAI* of this codon is relatively low and the model is sensitive to this value. Thus, we replaced the W*_i_* of this codon by mean *tAI* of this codons over all possible changes (+−0.5) of *S_ij_* -values.

From W*_i_* we obtain *p_i_*, which is the probability that a tRNA will be coupled to the codon
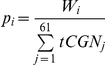
The expected time on codon 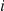 is 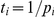.

The expected time on a site is the sum of times of all the codons in the site.

### Computing the Bottleneck

The bottleneck was defined as the slowest window in a gene. The time of a window in the sum of times corresponding to its codons; the size of a window is 15 codons (the results were robust to small changes in the size of the window).

### Running Times

Figure S17 depicts the running time of our model as a function of λ and site. As can be seen, when the site size is larger than 10 codons, for all λ the typical running time for a gene is less than 0.1 second.

### Real and Predicted Ribosome Density Profiles

Measurements of ribosome densities in *S. cerevisiae* at a resolution of single nucleotides were downloaded from [14]. For comparison to the predictions of various models the profiles were aligned to the beginning of the coding sequences (similarly to the way it was done in [7], [20]). We computed and plotted the mean densities in sites of size 15 codons for each of the profiles (measured and predicted).

### DTCO and DPCO - Estimating the Dependence of Genes on Codon Order in Terms of Translation Rate and Protein Abundance

To estimate the dependence of the translation rate of genes (at their ‘working point’) on codon order, DTCO, we performed the following steps:

1. Each mRNA transcript was randomly permuted (i.e., codons were randomly shuffled) 10 times. A library of permuted mRNA transcripts, associated with the original transcript, was thus generated and translation rates were computed for each transcript.
2. We then computed, for each gene separately, the standard deviation (*stdev*) for the set of rates obtained in stage 1.
3. For each gene, the *stdev* was normalized by the predicted translation rate of the gene (obtained from the un-permuted mRNA transcript).

We call this quantity *DTCO* and we use it as a measure for the dependence of the translation rate on codon order.

To estimate the dependence of protein abundance on the codon order, DPCO, we performed the following steps:

1. The relation between protein abundance and translation rates seems linear on a log-log scale (Figure S18, S19, S20); thus, we inferred a liner regressor of the log of protein abundance from the log of the predicted translation rate.
2. For each gene, and for each permutation, protein abundance was estimated via the regressor in (1). The stdev of the PA distribution associated with each gene (*i.e.*, of the library of permuted transcripts) was then computed.
3. For each gene, the stdev of the predicted protein abundance was normalized by the protein abundance of the original (un-permuted) mRNA.

### Finding the ‘Working Point’ of a Gene

To compute the ‘working point's of genes in a certain organism we first found the 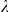 where the correlation between the mean predicted translation rate and protein abundance [16], [17], [62] is maximal. We computed the ratio (in percentages) between the mean genomic translation rate at this point and the mean maximal translation rate (for very large 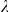); let *Q%* denote this value. (this value was 93%, 95%, and 99% in *S. cerevisiae*, *S. pombe*, and *E. coli* respectively)

The ‘working point’ of a gene in a certain organism is the 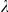 where the translation rate of the gene is *Q%* of its maximal translation rate.

### Analysis of the Data of Burgess-Brown et al

For each gene we computed the mean ratio between the synthetic version of the gene and its native version over 41 values of 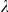 (between 0.0002 and 0.0094). The empirical p-value for the Spearman correlation is the probability that a random permutation of the two vectors will give higher correlation. It was computed by performing 100 such permutation and computing the Spearman correlation of each of them.

### The Statistical Test Used for Comparing the Genomic Ribosomal Densities Profile to the Predicted Profiles

The Wilcoxon rank test that we used is a paired non-parametric test where we compared (1) the vector of distances between the predictions of our model and the real data (a distance for each point); (2) the vector of distances between the predictions of tAI and the real data; (3) the vector of distances between the predictions of Zhang model and the real data. We compared (1) to (2) and (1) to (3) and checked the following statistical question: “is there improvement (in terms of the distance between predicted and real data points) when a more sophisticated model (*RFM*) is used instead of a less sophisticated one (*e.g.* the *tAI*).

### Jackknifing to Evaluate the Robustness of the Inferred Optimal Size of the Chunk

Jackknifing (see, e.g., [65]) was performed as described below.

Repeat 100 times:

1. Randomly choose 80% of the genes in *S. cerevisiae*.
2. Find the chunk size that gives the best correlation with protein abundance.

Report the number of cases (0–100) that we get C = 25.

The result confidence level was 100 demonstrating a very high confidence.

## Supporting Information

Figure S1 **Prediction of protein abundance by the various codon bias based predictors and by the ribosome flow model (RFM) for groups of genes with different levels of protein abundance in various organisms.** Prediction of protein abundance by the various codon bias based predictors of PA and by the ribosome flow model (RFM) for groups of genes with different levels of protein abundance in *S. cerevisiae* (A.), *E. coli* (B.), *S. pombe* (C.); all bins are of equal size. The RFM outperforms all the other predictors for lowly expressed genes (and in most of the bins) and has significant correlation with PA in all the bins.
(PDF)

Figure S2 **Correlation between protein abundance and the translation rate for various sizes of the translation site unit (** ***C*** **in** **Figure 1** **) in E. coli.**
(PDF)

Figure S3 **Correlation between protein abundance and the translation rate for various sizes of the translation site unit (** ***C*** **in** **Figure 1** **) in S. pombe.**
(PDF)

Figure S4 **The** ***RFM*** **predicts the genomic profile of ribosome densities in starvation better than the** ***tAI*** **model or the predictor of Zhang** ***et al*** **.** All the figures were normalized to have the same mean.
(PDF)

Figure S5
**The relation between** 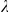 **(the number of available ribosomes in the cell), mean of the translation rate (number of proteins per time unit), and the mean ribosome density in** ***E. coli*** **.**
(PDF)

Figure S6
**The relation between** 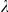 **(the number of available ribosomes in the cell), mean of the translation rate (number of proteins per time unit), and the mean ribosome density in** ***Human liver*** **.**
(PDF)

Figure S7
**The relation between** 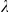 **(the number of available ribosomes in the cell), mean of the translation rate (number of proteins per time unit), and the mean ribosome density in** ***S. pombe*** **.**
(PDF)

Figure S8 **Dot plot – log protein abundance** ***vs.*** **initiation rate in** ***S. cerevisiae*** **.**
(PDF)

Figure S9 **Dot plot – log protein abundance** ***vs.*** **initiation rate in** ***S. pombe*** **.**
(PDF)

Figure S10 **Dot plot – log protein abundance** ***vs.*** **initiation rate in** ***E. coli*** **.**
(PDF)

Figure S11 **Dot plot – ribosome density** ***vs.*** **initiation rate in** ***S. pombe*** *.*
(PDF)

Figure S12 **Dot plot – ribosome density** ***vs.*** **initiation rate in** ***S. cerevisiae*** **.**
(PDF)

Figure S13 **Dot plot – ribosome density** ***vs.*** **initiation rate in** ***E. coli*** **.**
(PDF)

Figure S14 **Dot plot – log protein abundance** ***vs.*** **ribosome density in** ***S. cerevisiae*** **.**
(PDF)

Figure S15 **Dot plot – log protein abundance** ***vs.*** **ribosome density in** ***S. pombe*** **.**
(PDF)

Figure S16 **Dot plot – log protein abundance** ***vs.*** **ribosome density in** ***E. coli*** **.**
(PDF)

Figure S17 **Mean running time (in seconds) for computing the translation rate of the RFM as a function of and size of the site.**
(PDF)

Figure S18 **Dot plot – log protein abundance** ***vs.*** **log predicted translation rate in** ***S. pombe*** **.**
(PDF)

Figure S19 **Dot plot – log protein abundance** ***vs.*** **log predicted translation rate in** ***E. coli*** **.**
(PDF)

Figure S20 **Dot plot – log protein abundance** ***vs.*** **log predicted translation rate in** ***S. cerevisiae*** **.**
(PDF)

Figure S21 **Correlation of the** ***tAI*** **and the RFM and with protein abundance given mRNA levels for groups of genes with different levels of protein in** ***E. coli*** **.** All bins are of equal size.
(PDF)

Figure S22 **Correlation of the tAI and the RFM with protein abundance given mRNA levels for groups of genes with different levels of protein in** ***S. pombe*** **.** All bins are of equal size.
(PDF)

Figure S23 **Correlation of the tAI and the RFM with protein abundance multiplied by mRNA levels for groups of genes with different levels of protein in** ***S. pombe*** **.** All bins are of equal size.
(PDF)

Figure S24 **Correlation of the tAI and the RFM with protein abundance given mRNA levels for groups of genes with different levels of protein in** ***S. cerevisiae*** **.** All bins are of equal size.
(PDF)

Figure S25 **Correlation of the tAI and the RFM with protein abundance multiplies by the mRNA levels for groups of genes with different levels of protein in** ***S. cerevisiae*** **.** All bins are of equal size.
(PDF)

Figure S26 **Profiles of tAI of cytosolic and mitochondrial ribosomal proteins in** ***S. cerevisiae*** **.**
(PDF)

Figure S27 **Profiles of tAI of highly expressed genes and lowly expressed genes in** ***S. cerevisiae*** **.** Close to the 5′ end of the genes there is a region with slower speed. This region is more prominent in highly expressed genes.
(PDF)

Text S1 **The justification for using the tAI and the RFM as an predictor of the co-adaptation between codon bias and tRNA pool.**
(PDF)

Text S2 **Endogenous genes in** ***S. cerevisiae*** **,** ***S. pombe*** **, and** ***E. coli*** **: correlation of the predicted rates with protein abundance given mRNA levels and the correlation of the predicted rate multiplies by the mRNA levels with protein abundance.**
(PDF)

Text S3 **The predictions of the tAI and translation efficiency profiles of genes.**
(PDF)

Text S4 **The genomic rate of** ***abortion*** **of ribosomes has power law decay.**
(PDF)

Text S5 **The initiation rates used in this study are robust and not over-fitted.**
(PDF)

Text S6 **Tissue-specific translation rates in Human.**
(PDF)
