# Supplementary figures and images for: Genome-Scale Analysis of Translation Elongation with a Ribosome Flow Model
Source: PLoS Comput Biol. 2011 Sep 1;7(9):e1002127. doi: 10.1371/journal.pcbi.1002127 (PMC3164701; doi:10.1371/journal.pcbi.1002127)

A.

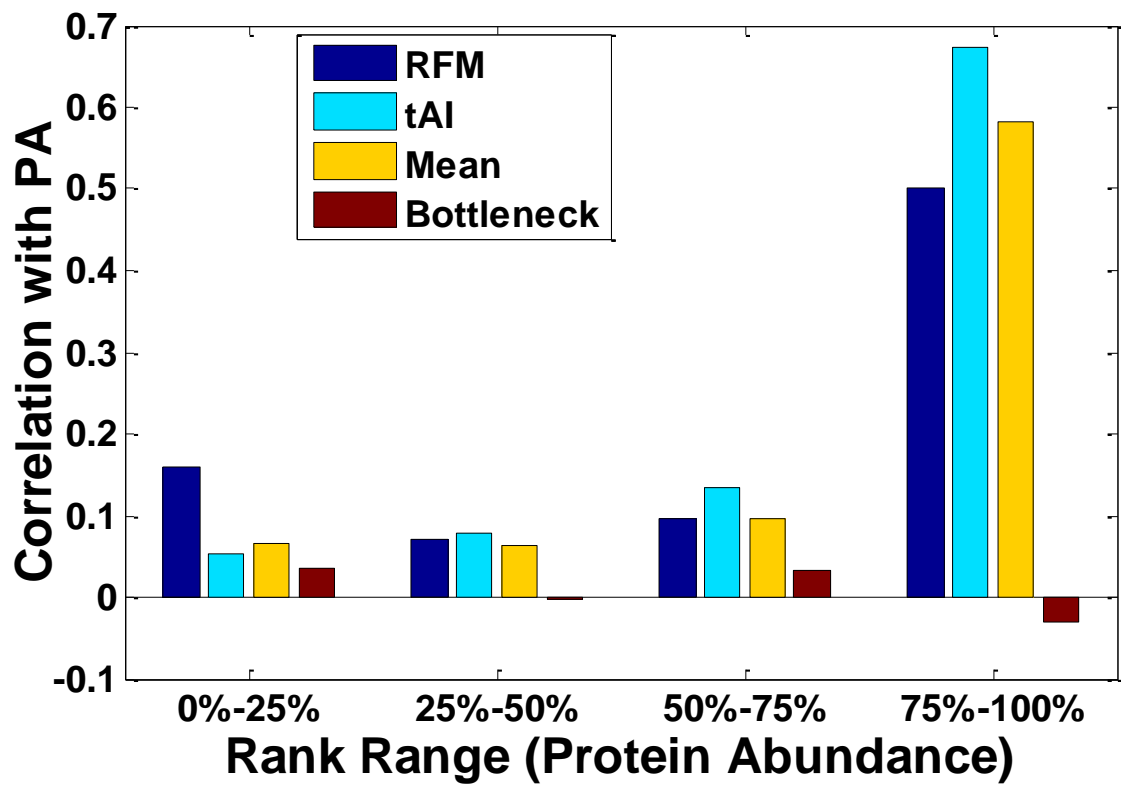

B.

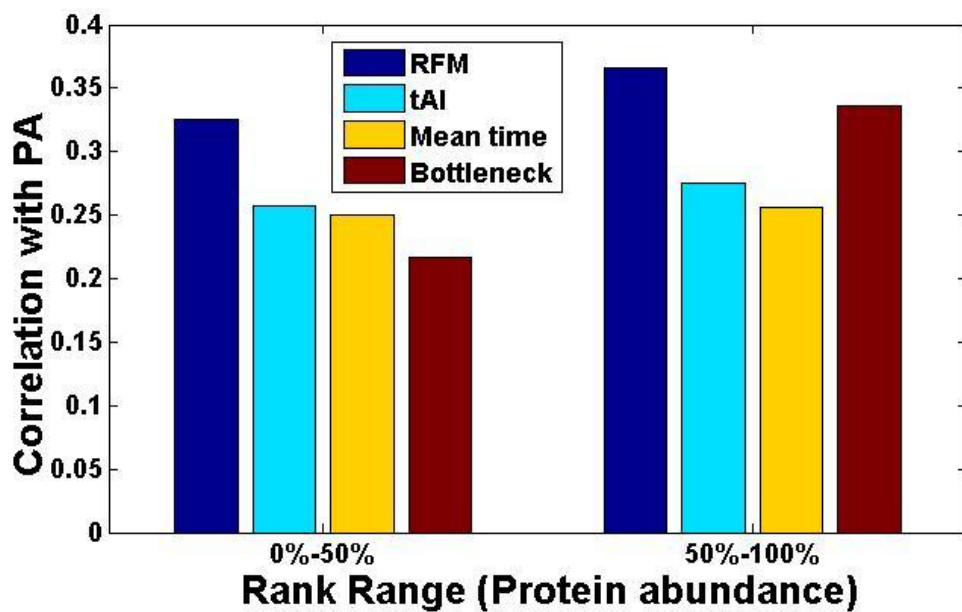

C.

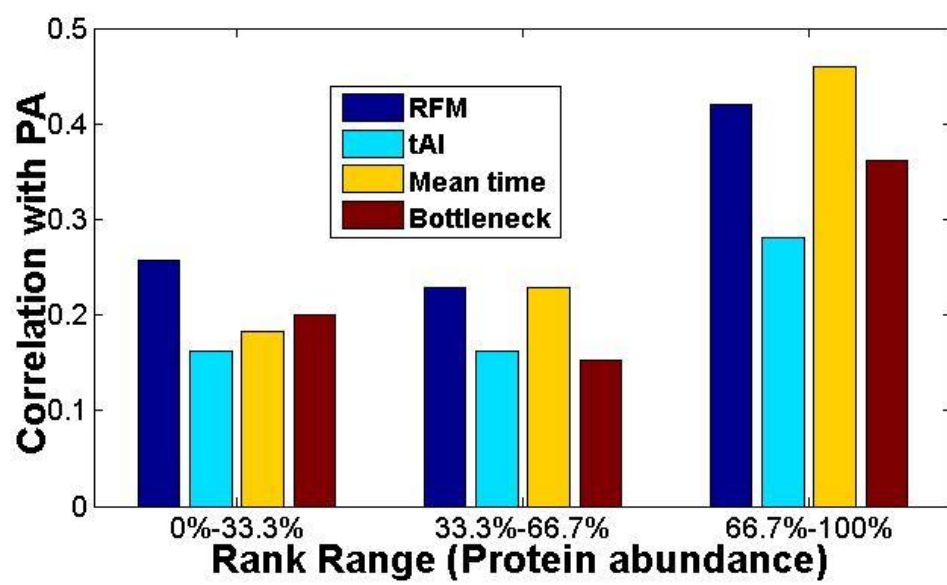

Supplement: Figure S1 — Prediction of protein abundance by the various codon bias based predictors and by the ribosome flow model (RFM) for groups of genes with different levels of protein abundance in various organisms. Prediction of protein abundance by the various codon bias based predictors of PA and by the ribosome flow model (RFM) for groups of genes with different levels of protein abundance in S. cerevisiae (A.), E. coli (B.), S. pombe (C.); all bins are of equal size. The RFM outperforms all the other predictors for lowly expressed genes (and in most of the bins) and has significant correlation with PA in all the bins. (PDF) [file pcbi.1002127.s001.pdf]

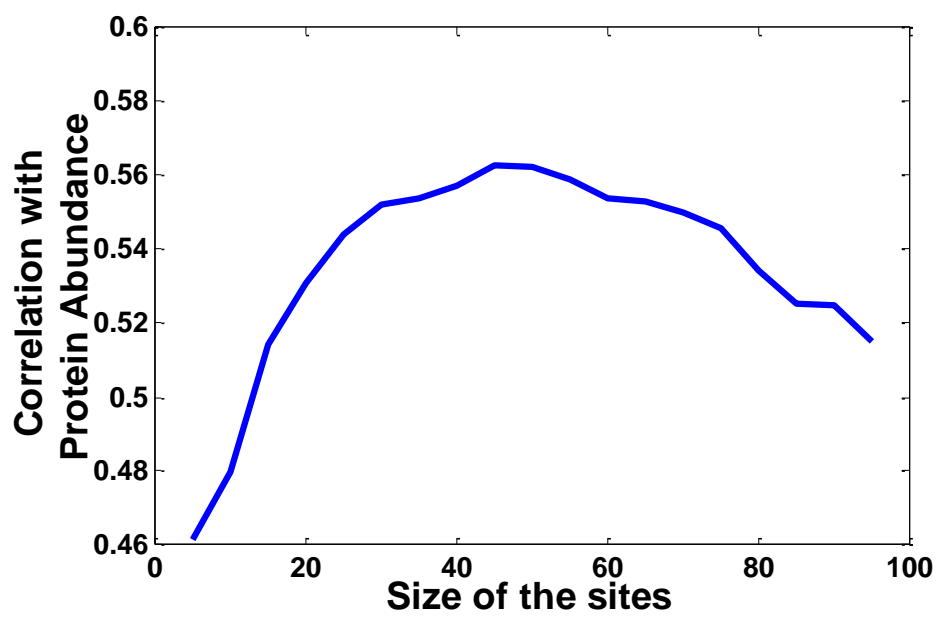

Supplement: Figure S2 — Correlation between protein abundance and the translation rate for various sizes of the translation site unit ( C in Figure 1 ) in E. coli. (PDF) [file pcbi.1002127.s002.pdf]

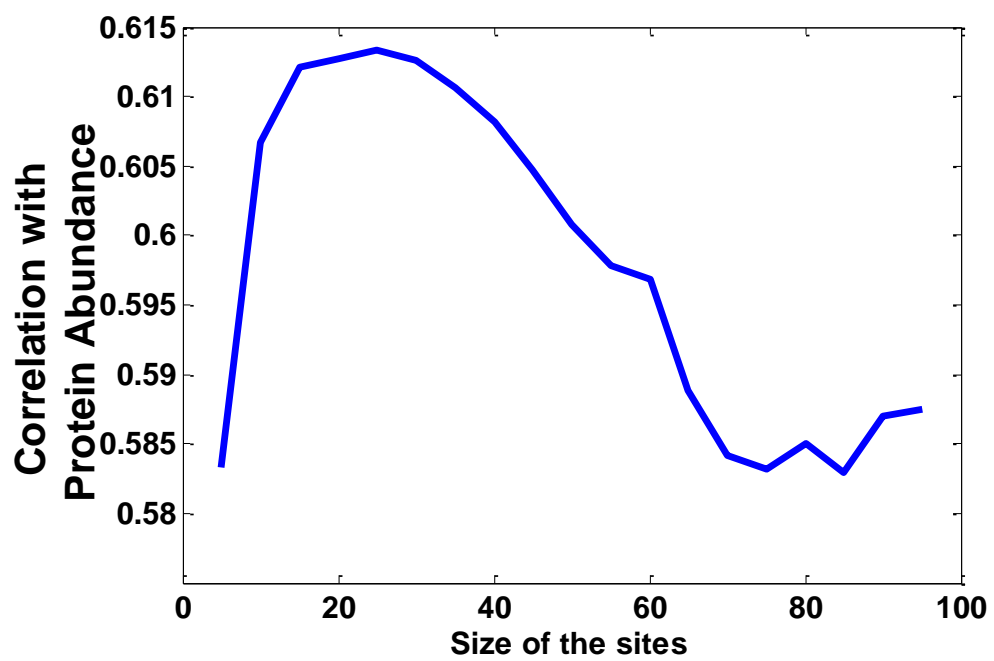

Supplement: Figure S3 — Correlation between protein abundance and the translation rate for various sizes of the translation site unit ( C in Figure 1 ) in S. pombe. (PDF) [file pcbi.1002127.s003.pdf]

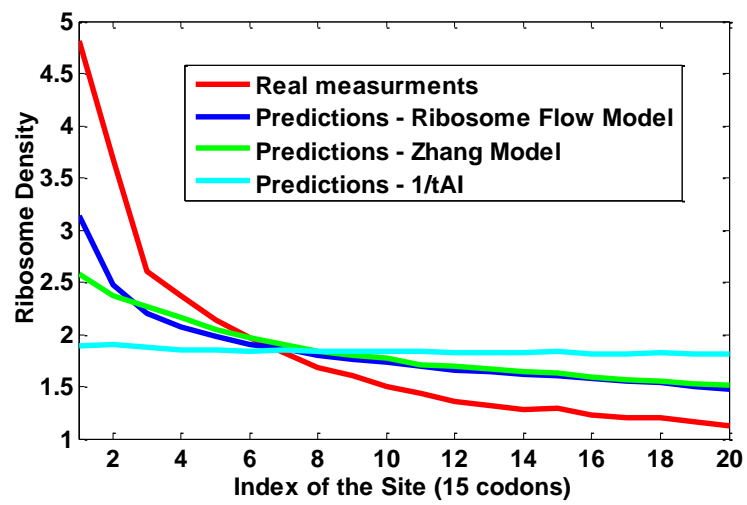

Supplement: Figure S4 — The RFM predicts the genomic profile of ribosome densities in starvation better than the tAI model or the predictor of Zhang et al . All the figures were normalized to have the same mean. (PDF) [file pcbi.1002127.s004.pdf]

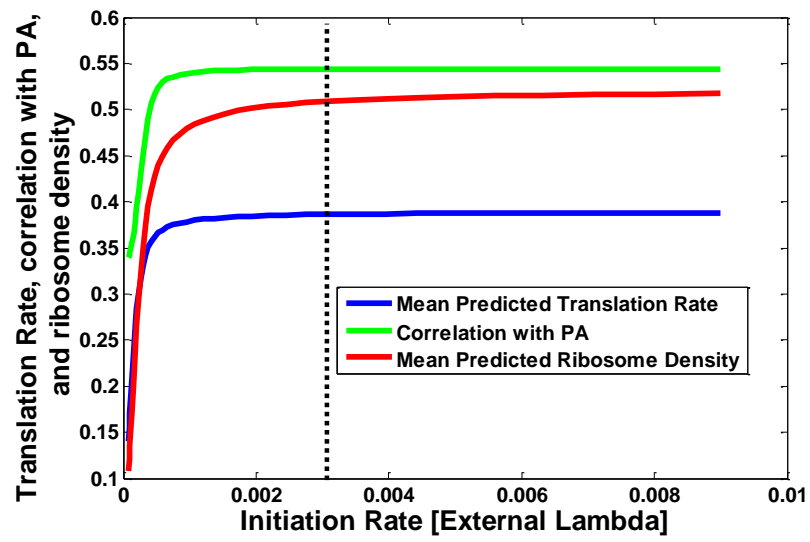

Supplement: Figure S5 — The relation between (the number of available ribosomes in the cell), mean of the translation rate (number of proteins per time unit), and the mean ribosome density in E. coli . (PDF) [file pcbi.1002127.s005.pdf]

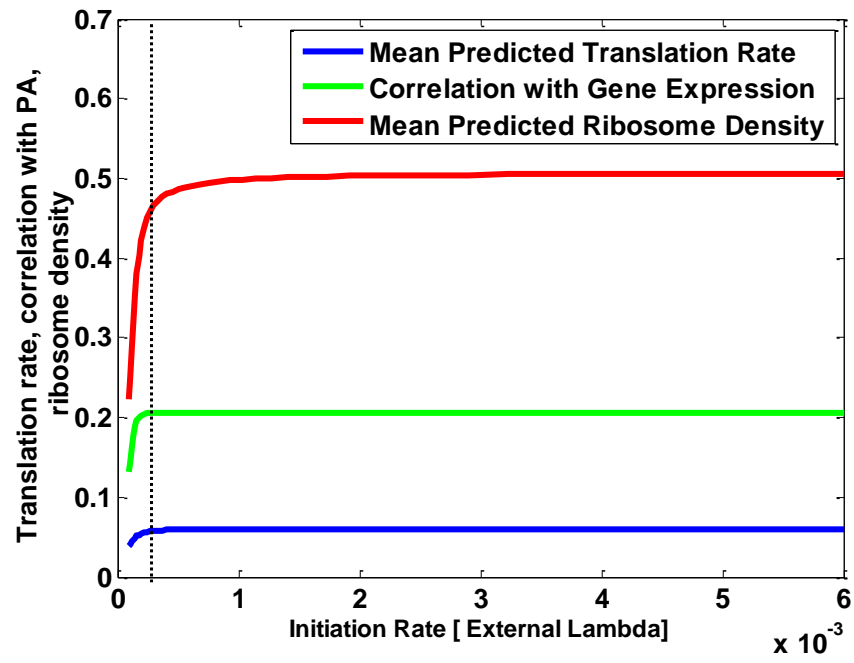

Supplement: Figure S6 — The relation between (the number of available ribosomes in the cell), mean of the translation rate (number of proteins per time unit), and the mean ribosome density in Human liver . (PDF) [file pcbi.1002127.s006.pdf]

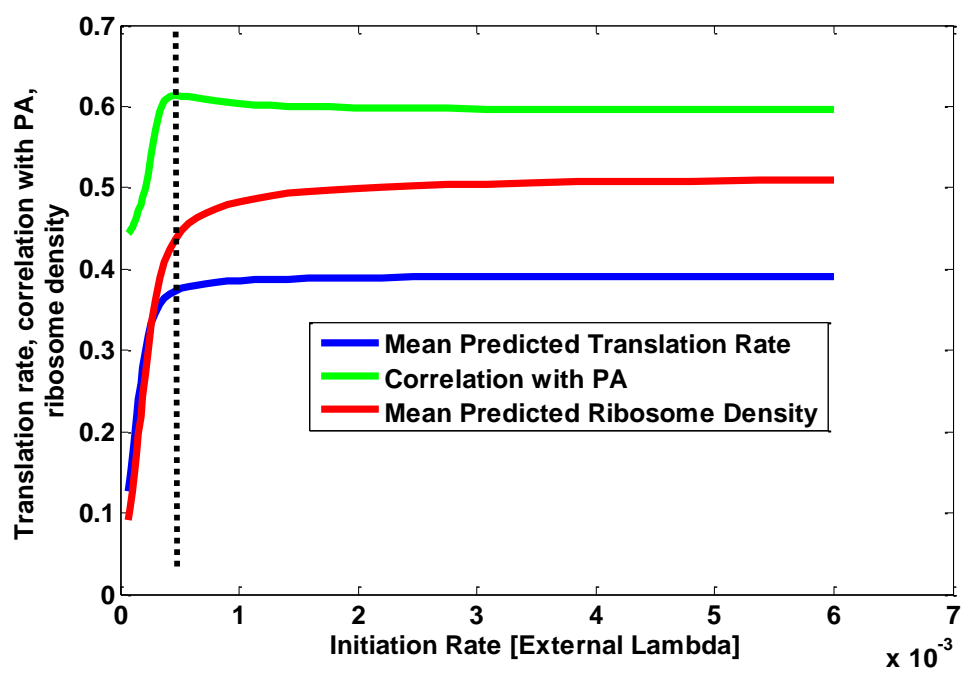

Supplement: Figure S7 — The relation between (the number of available ribosomes in the cell), mean of the translation rate (number of proteins per time unit), and the mean ribosome density in S. pombe . (PDF) [file pcbi.1002127.s007.pdf]

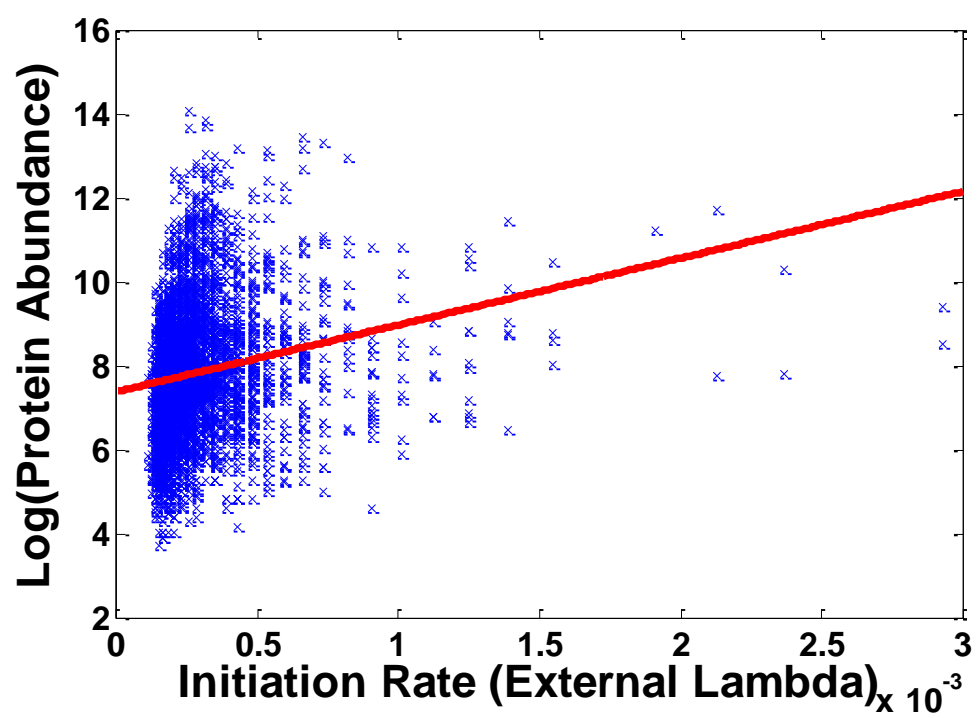

Supplement: Figure S8 — Dot plot – log protein abundance vs. initiation rate in S. cerevisiae . (PDF) [file pcbi.1002127.s008.pdf]

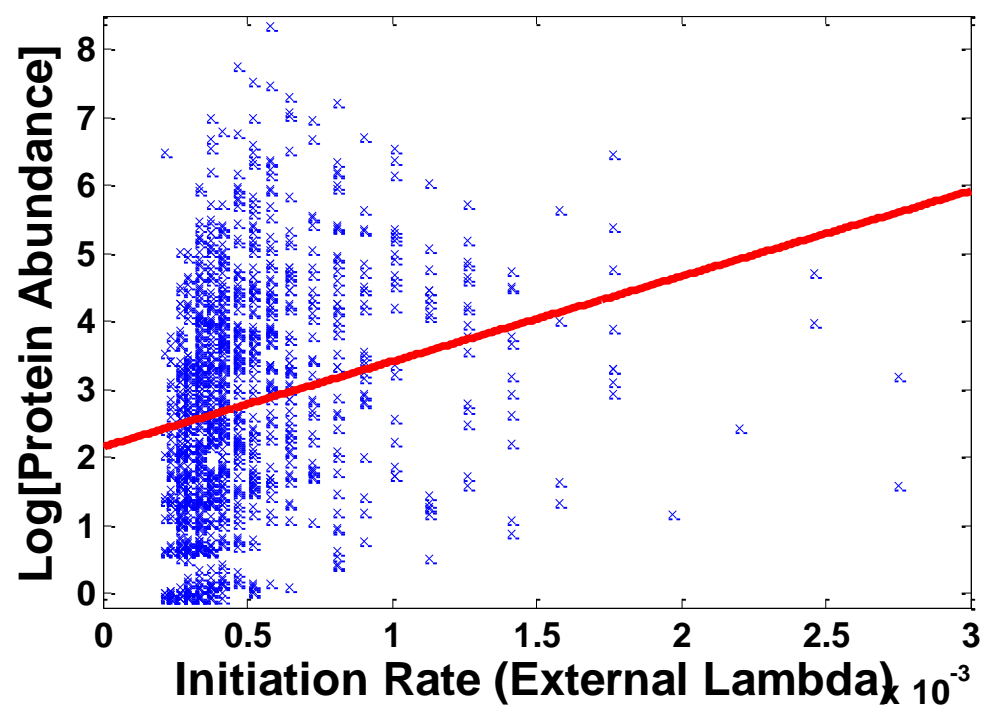

Supplement: Figure S9 — Dot plot – log protein abundance vs. initiation rate in S. pombe . (PDF) [file pcbi.1002127.s009.pdf]

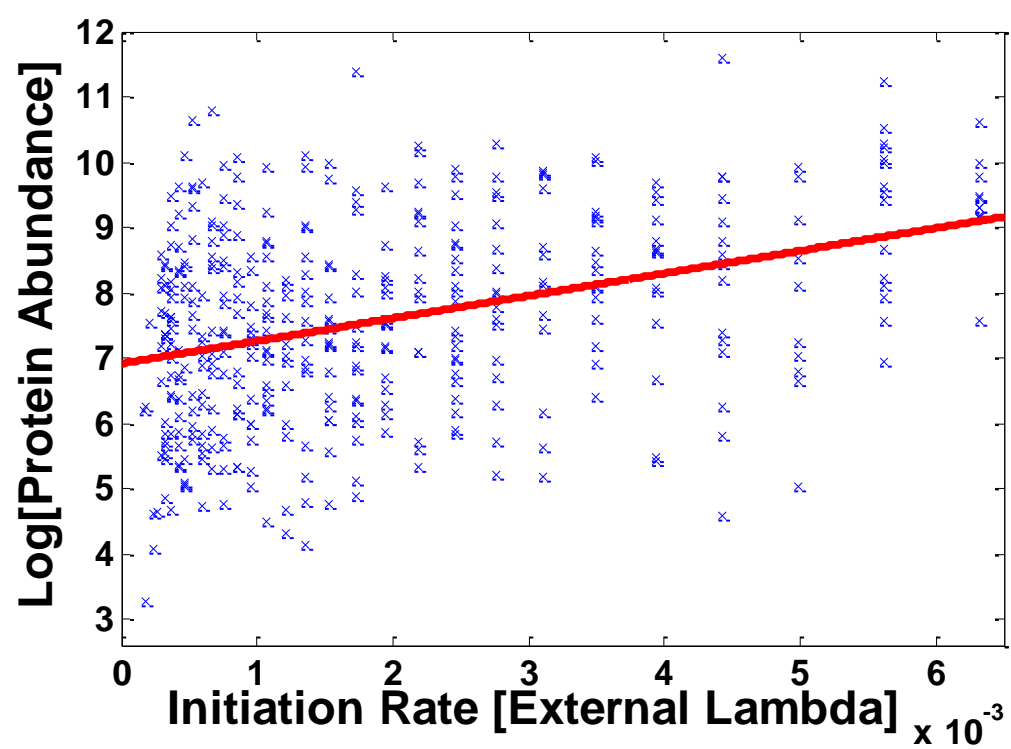

Supplement: Figure S10 — Dot plot – log protein abundance vs. initiation rate in E. coli . (PDF) [file pcbi.1002127.s010.pdf]

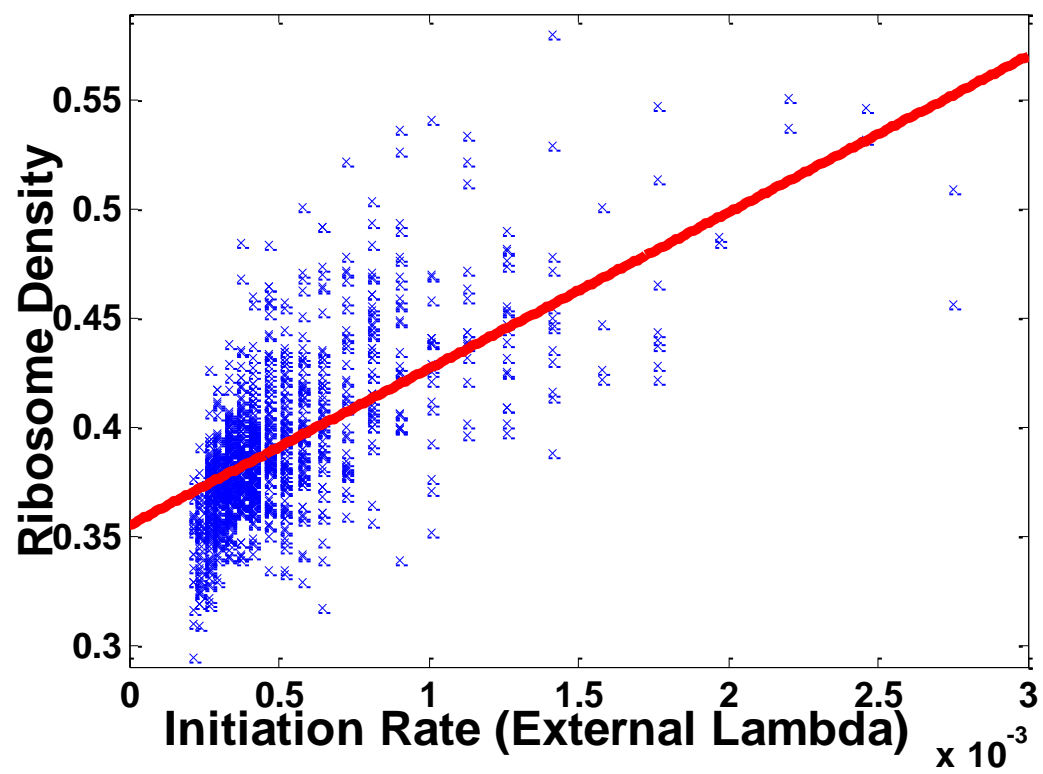

Supplement: Figure S11 — Dot plot – ribosome density vs. initiation rate in S. pombe . (PDF) [file pcbi.1002127.s011.pdf]

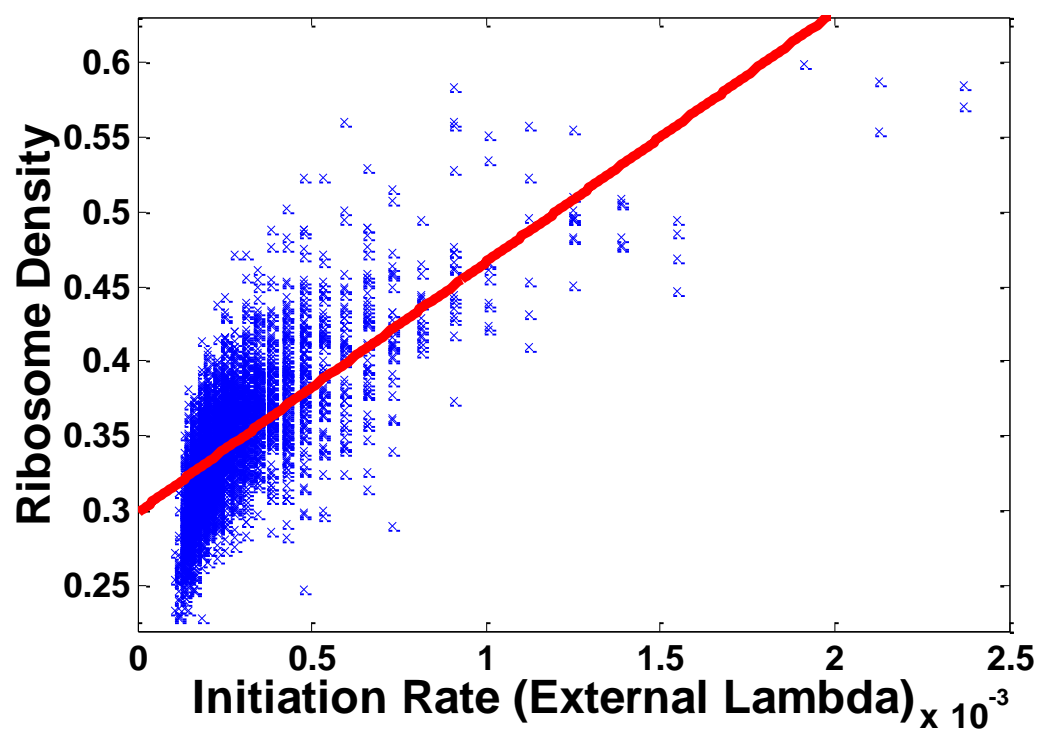

Supplement: Figure S12 — Dot plot – ribosome density vs. initiation rate in S. cerevisiae . (PDF) [file pcbi.1002127.s012.pdf]

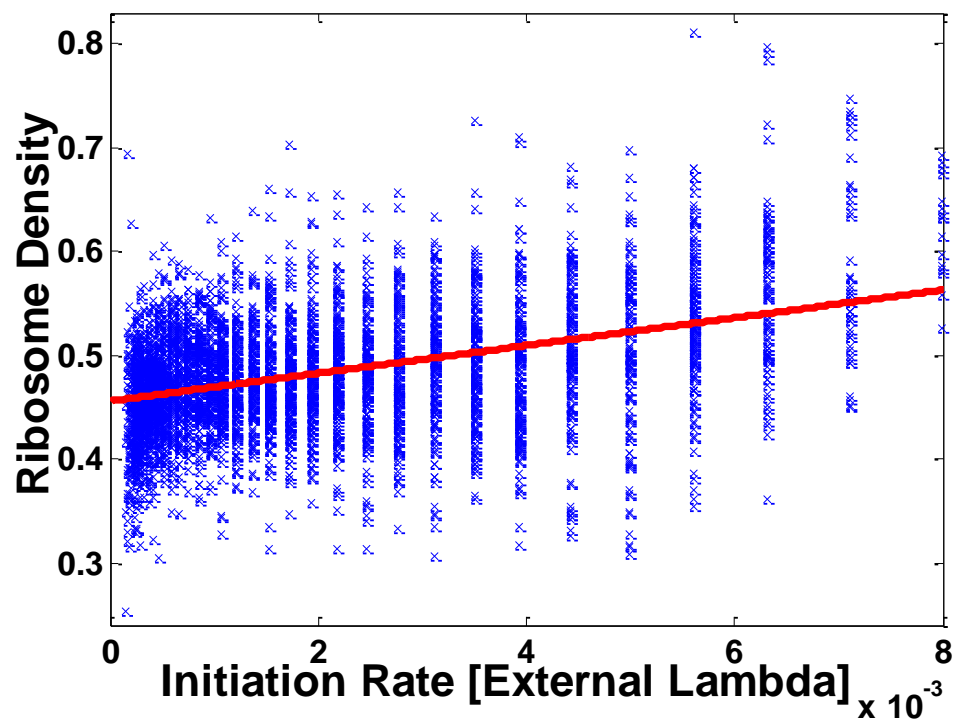

Supplement: Figure S13 — Dot plot – ribosome density vs. initiation rate in E. coli . (PDF) [file pcbi.1002127.s013.pdf]

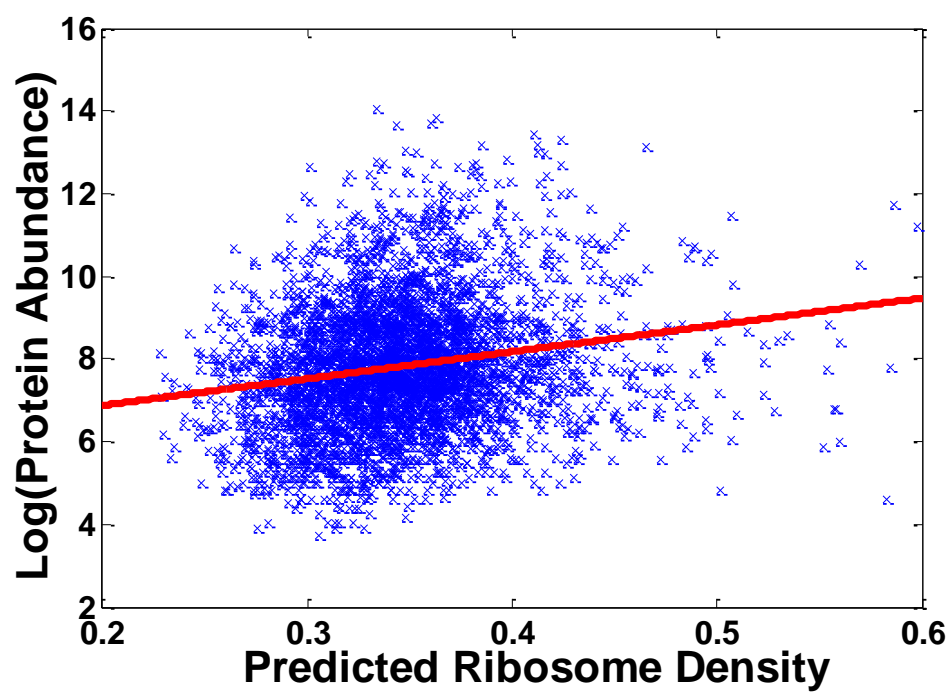

Supplement: Figure S14 — Dot plot – log protein abundance vs. ribosome density in S. cerevisiae . (PDF) [file pcbi.1002127.s014.pdf]

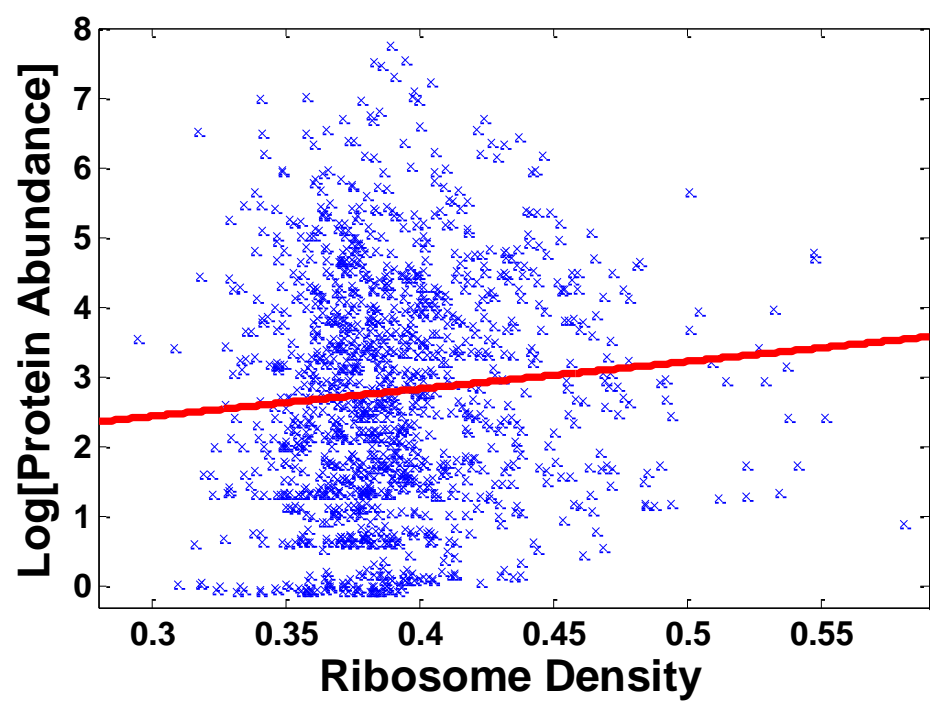

Supplement: Figure S15 — Dot plot – log protein abundance vs. ribosome density in S. pombe . (PDF) [file pcbi.1002127.s015.pdf]

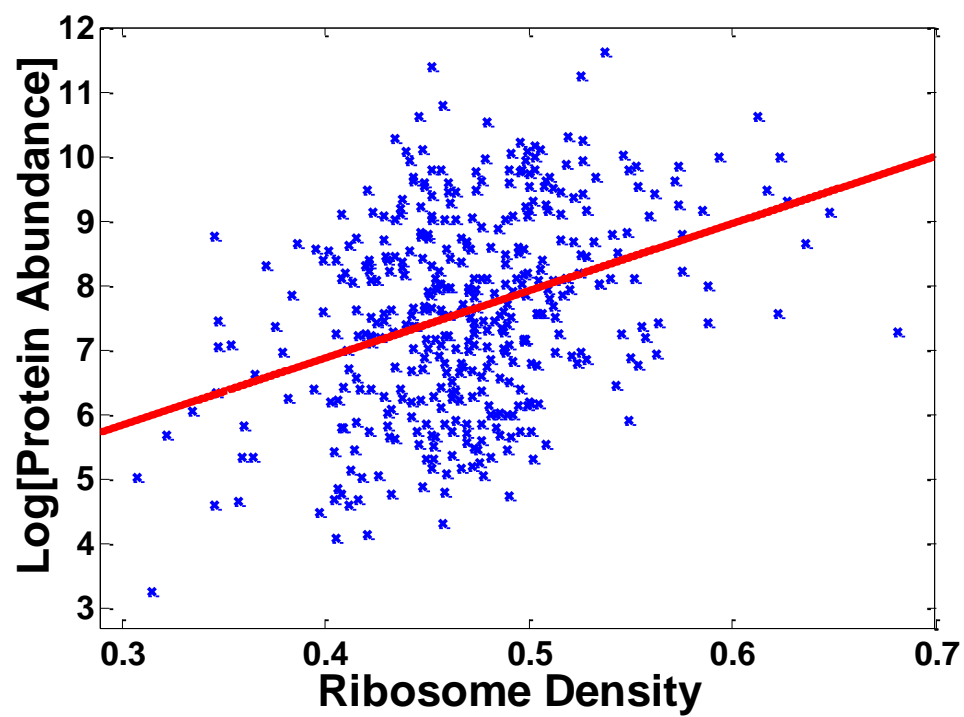

Supplement: Figure S16 — Dot plot – log protein abundance vs. ribosome density in E. coli . (PDF) [file pcbi.1002127.s016.pdf]

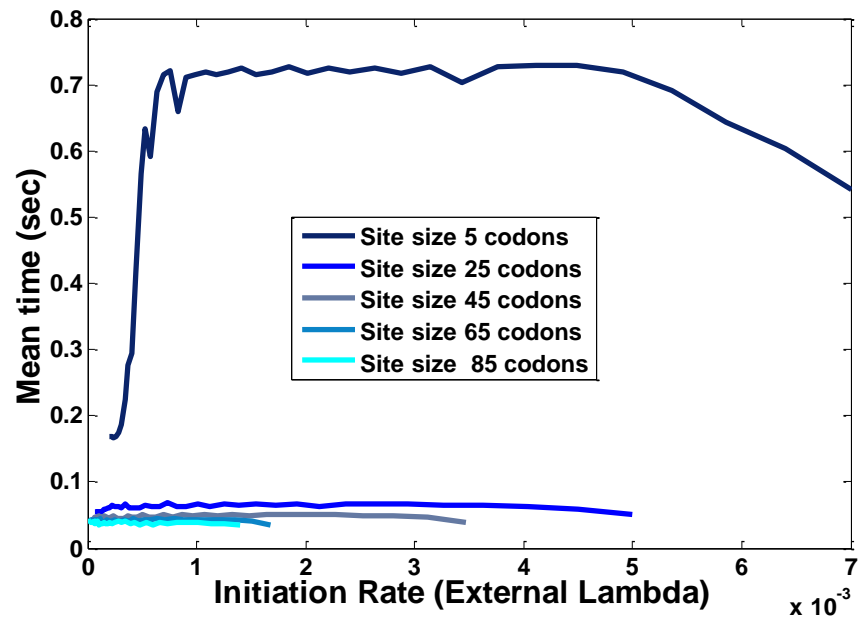

Supplement: Figure S17 — Mean running time (in seconds) for computing the translation rate of the RFM as a function of and size of the site. (PDF) [file pcbi.1002127.s017.pdf]

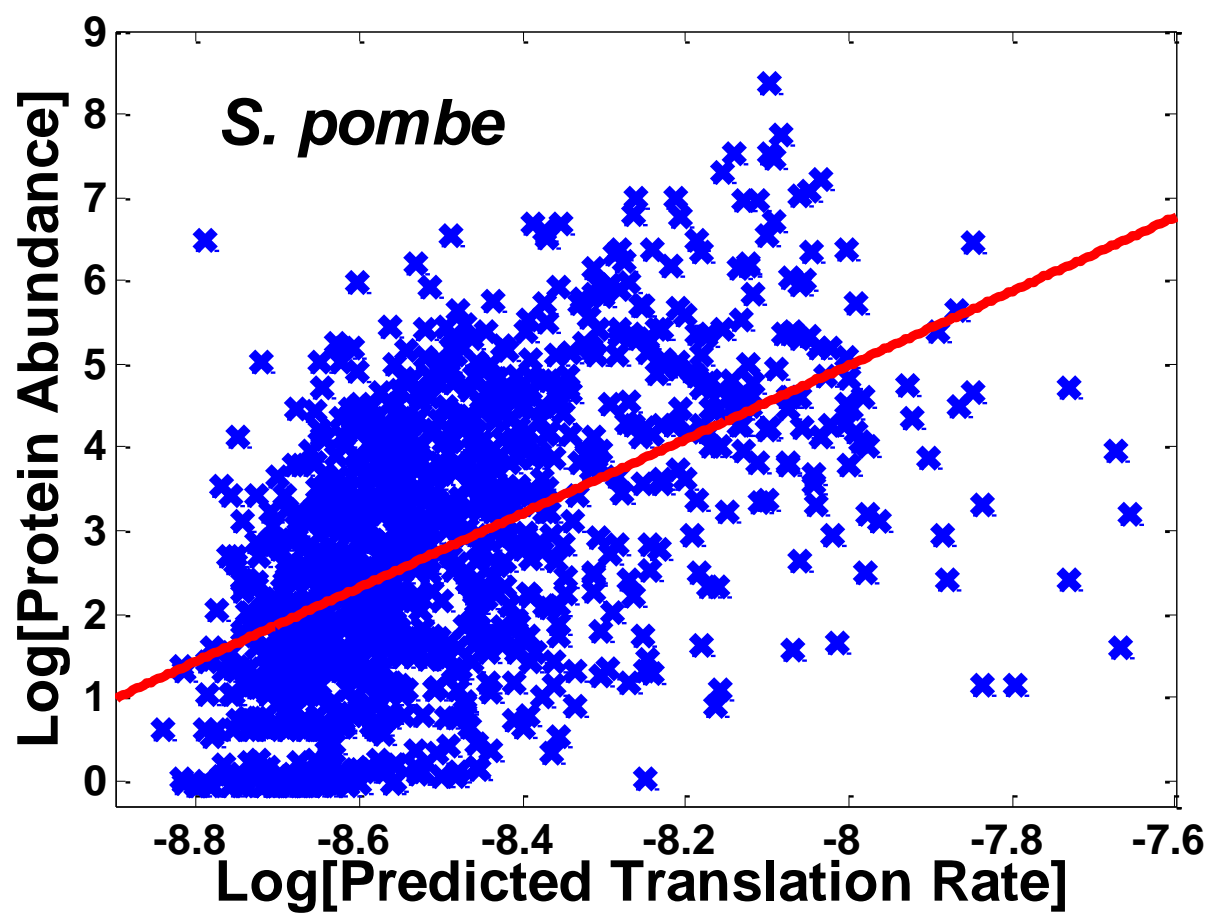

Supplement: Figure S18 — Dot plot – log protein abundance vs. log predicted translation rate in S. pombe . (PDF) [file pcbi.1002127.s018.pdf]

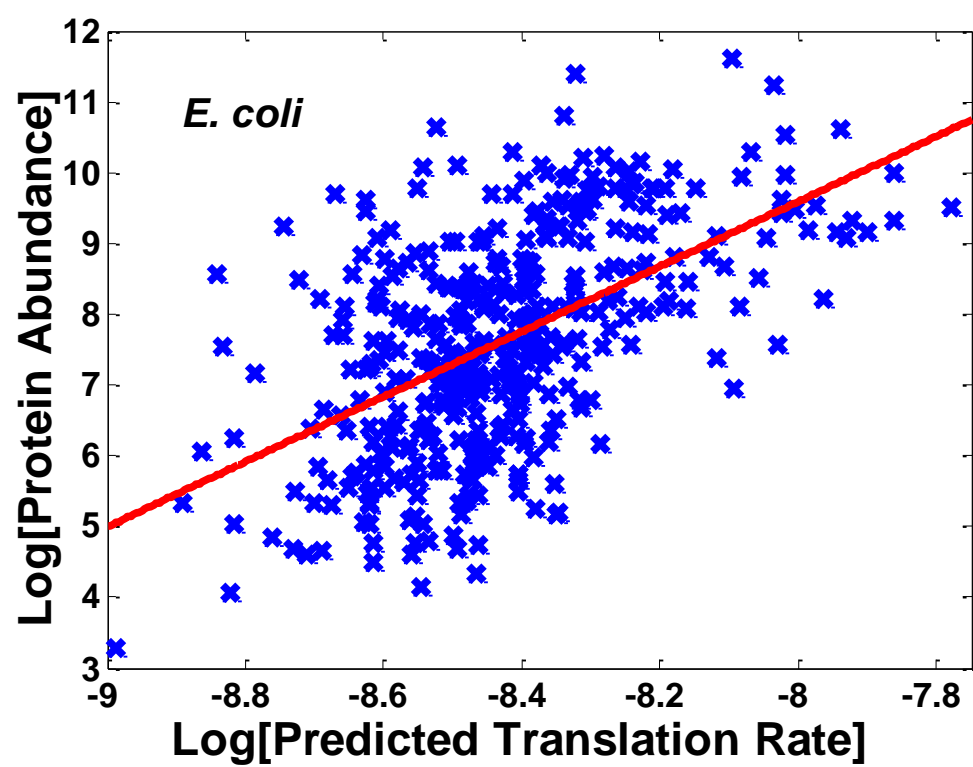

Supplement: Figure S19 — Dot plot – log protein abundance vs. log predicted translation rate in E. coli . (PDF) [file pcbi.1002127.s019.pdf]

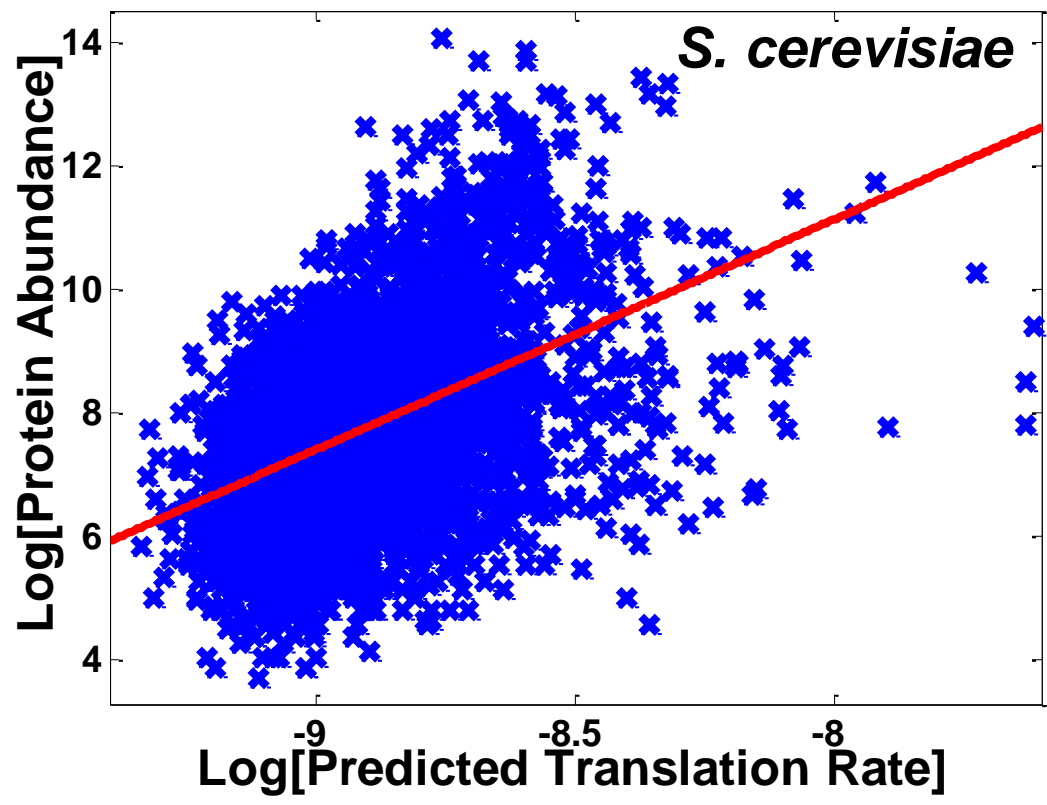

Supplement: Figure S20 — Dot plot – log protein abundance vs. log predicted translation rate in S. cerevisiae . (PDF) [file pcbi.1002127.s020.pdf]

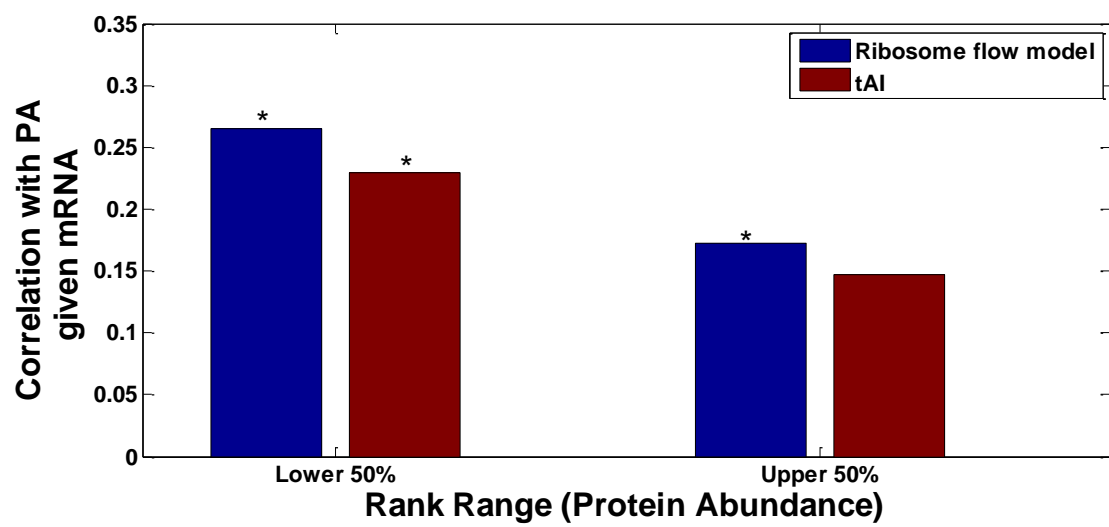

Supplement: Figure S21 — Correlation of the tAI and the RFM and with protein abundance given mRNA levels for groups of genes with different levels of protein in E. coli . All bins are of equal size. (PDF) [file pcbi.1002127.s021.pdf]

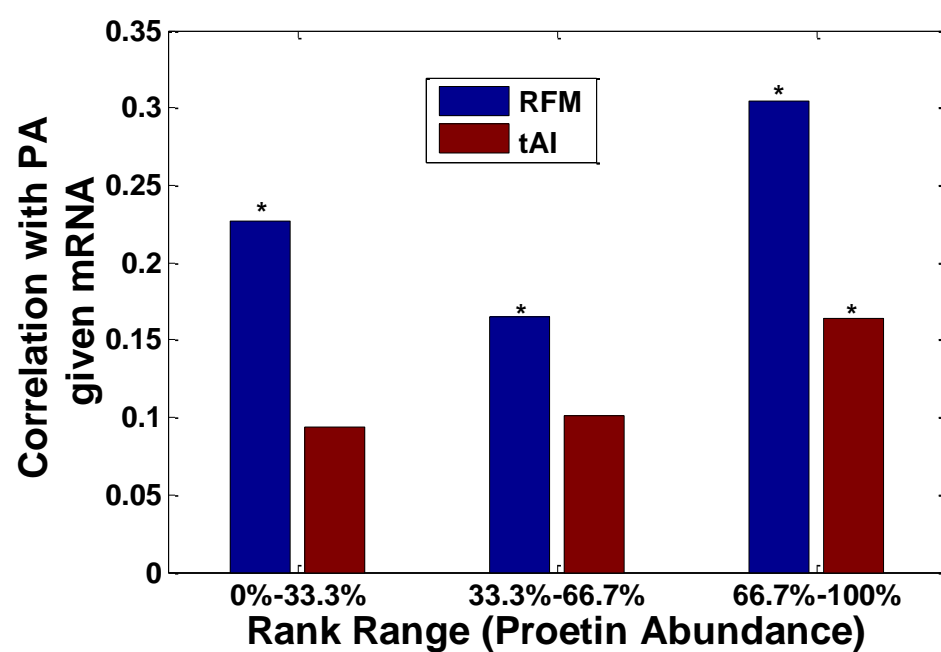

Supplement: Figure S22 — Correlation of the tAI and the RFM with protein abundance given mRNA levels for groups of genes with different levels of protein in S. pombe . All bins are of equal size. (PDF) [file pcbi.1002127.s022.pdf]

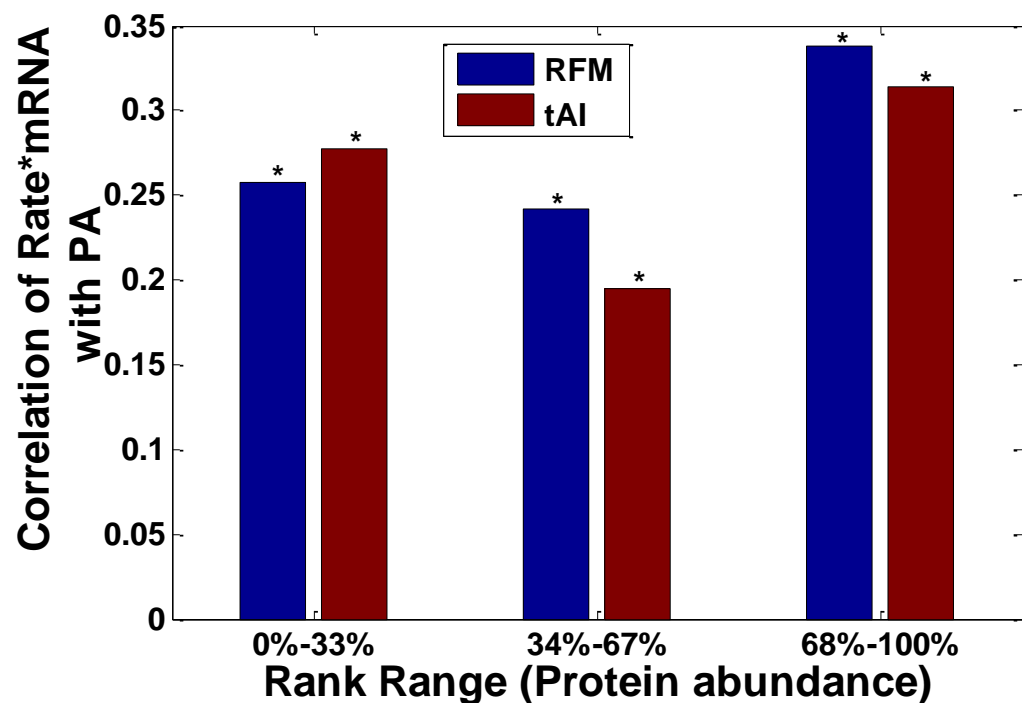

Supplement: Figure S23 — Correlation of the tAI and the RFM with protein abundance multiplied by mRNA levels for groups of genes with different levels of protein in S. pombe . All bins are of equal size. (PDF) [file pcbi.1002127.s023.pdf]

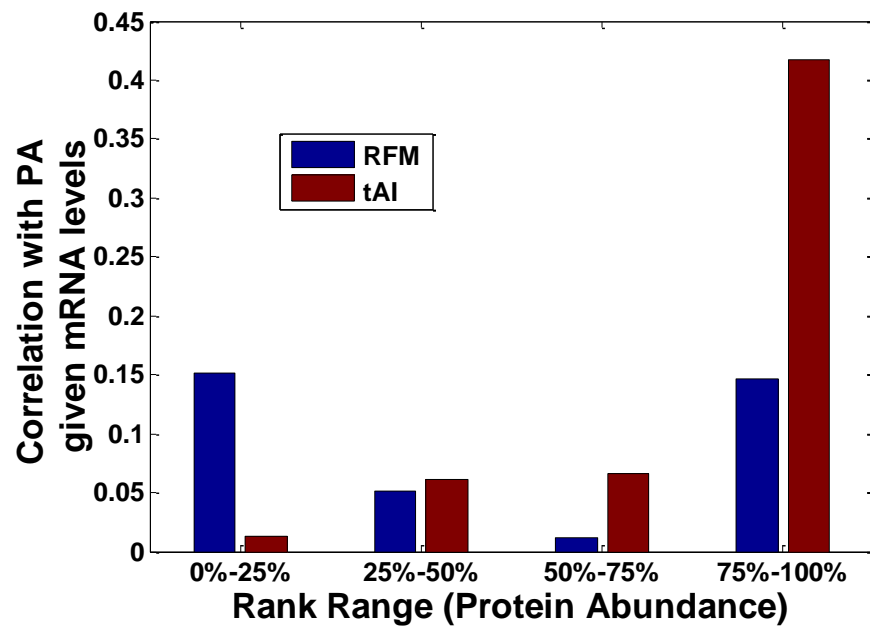

Supplement: Figure S24 — Correlation of the tAI and the RFM with protein abundance given mRNA levels for groups of genes with different levels of protein in S. cerevisiae . All bins are of equal size. (PDF) [file pcbi.1002127.s024.pdf]

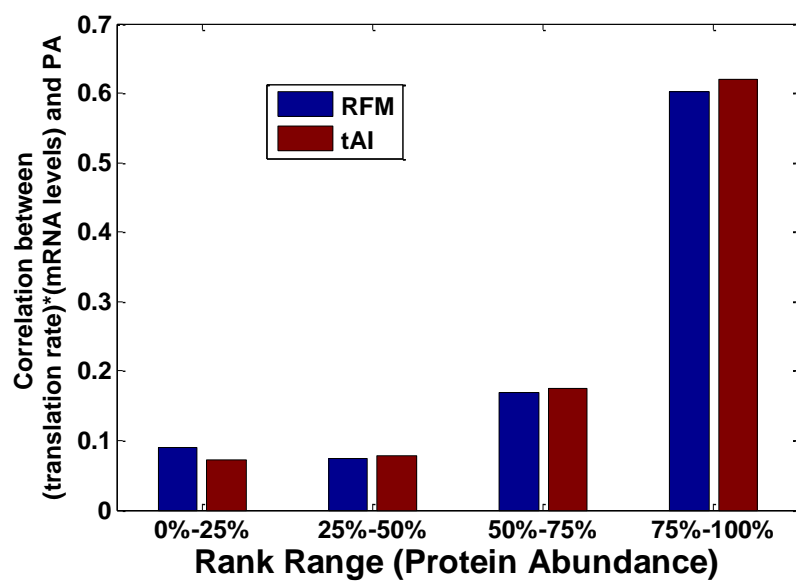

Supplement: Figure S25 — Correlation of the tAI and the RFM with protein abundance multiplies by the mRNA levels for groups of genes with different levels of protein in S. cerevisiae . All bins are of equal size. (PDF) [file pcbi.1002127.s025.pdf]

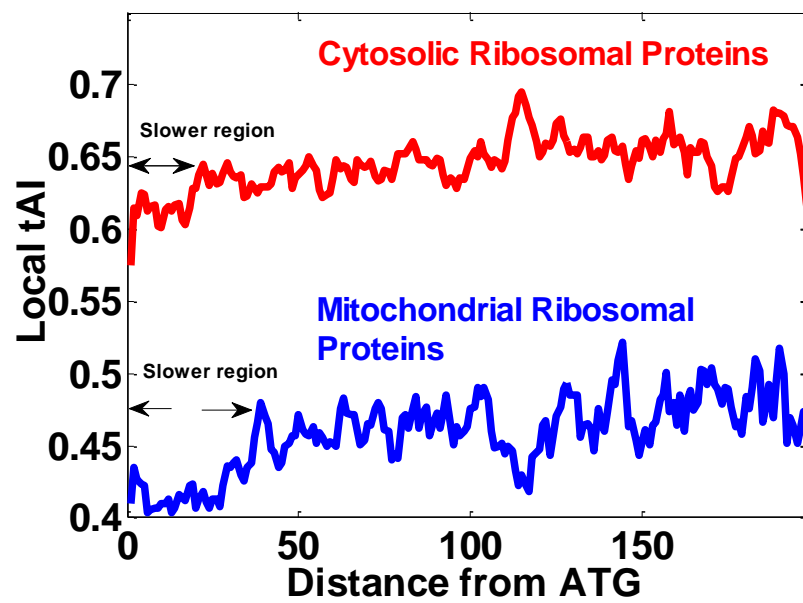

Supplement: Figure S26 — Profiles of tAI of cytosolic and mitochondrial ribosomal proteins in S. cerevisiae . (PDF) [file pcbi.1002127.s026.pdf]

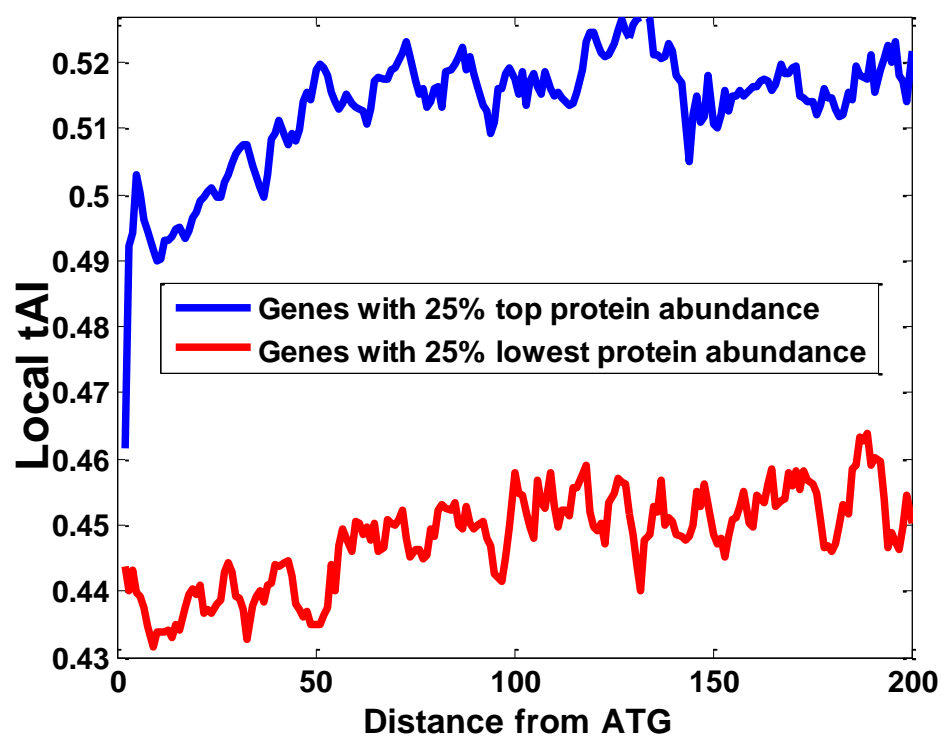

Supplement: Figure S27 — Profiles of tAI of highly expressed genes and lowly expressed genes in S. cerevisiae . Close to the 5′ end of the genes there is a region with slower speed. This region is more prominent in highly expressed genes. (PDF) [file pcbi.1002127.s027.pdf]
