# Supplementary material for: Genome-Scale Analysis of Translation Elongation with a Ribosome Flow Model
Source: PLoS Comput Biol. 2011 Sep 1;7(9):e1002127. doi: 10.1371/journal.pcbi.1002127 (PMC3164701; doi:10.1371/journal.pcbi.1002127)
Supplement: Text S1 — The justification for using the tAI and the RFM as an predictor of the co-adaptation between codon bias and tRNA pool. (PDF) [file pcbi.1002127.s028.pdf]

Text S1 1: the justification for using the tAI and the RFM as an predictor of the co-adaptation between codon bias and tRNA pool

The tAI and the *RFM* are based on the genomic tRNA copy number (tGCN; when the expression levels of the tRNAs is unknown) as a surrogate measure for the cellular abundances of tRNAs; it is justified by several observations.

First, in the past, in many organisms, it has been observed that the *in vivo* concentration of a tRNA bearing a certain anticodon is highly proportional to the number of gene copies coding for this tRNA type. Specifically, in *S. cerevisiae* a correlation of r=0.91 [1] was reported. In *B. subtilis*, a correlation of 0.86 between tRNA copy number and tRNA abundance was reported [2]. Similarly, previous papers reported about significant correlation between genomic tRNA copy number and tRNA abundance in *E. coli* [3,4]. A related interesting result is the analysis of [5] who measured the translation rate of two glutamate codons: GAA and GAG. They found them to have a threefold difference in translation rate (21.6 and 6.4 codons per second, respectively). Remarkably, the w_i of these codons, which is based on the tRNA pool and affinity of codon-anti-codon coupling and is the basis for the tAI calculation, captures the ratio of translation rate between the two codons. Calculating w_i values for *E. coli* we found that the ratio between the w_i of GAA and GAG is 3.125 (0.5/0.16) as compared to the 3.34 reported in the experiments (21.4/6.4). This result suggests that there is a direct relation between the adaptation of a codon to the tRNA pool, based on the genomic tRNA copy number, and the time it takes to translate it.

Second, a recent study showed that in *S. cerevisiae* the promoters of many of the tRNA genes have a low predicted affinity to the nucleosome, suggesting a constitutive expression with little transcriptional regulation capacity [6]. Thus, for fully sequenced genomes, the relative concentrations of the various tRNAs in the cell, and therefore the optimality of the various codons in terms of translation, can be approximated using the respective tRNA gene copy numbers in the genome. Additionally, as we show in this paper, measures that are based on tRNA copy number highly correlated ro protein expression levels (see also [7,8]). It was found that even among genes with similar transcript levels, higher tAI often corresponds to higher protein abundance [7].

1. Percudani R, Pavesi A, Ottonello S (1997) Transfer RNA gene redundancy and translational selection in Saccharomyces cerevisiae. J Mol Biol 268: 322-330.
2. Kanaya S, Yamada Y, Kudo Y, Ikemura T (1999) Studies of codon usage and tRNA genes of 18 unicellular organisms and quantification of Bacillus subtilis tRNAs: gene expression level and species-specific diversity of codon usage based on multivariate analysis. Gene 238: 143-155.
3. Ikemura T (1981) Correlation between the abundance of Escherichia coli transfer RNAs and the occurrence of the respective codons in its protein genes: a proposal for a synonymous codon choice that is optimal for the E. coli translational system. J Mol Biol 151: 389-409.
4. Dong H, Nilsson L, Kurland CG (1996) Co-variation of tRNA abundance and codon usage in Escherichia coli at different growth rates. J Mol Biol 260: 649-663.
5. Sorensen MA, Pedersen S (1991) Absolute in vivo translation rates of individual codons in Escherichia coli. The two glutamic acid codons GAA and GAG are translated with a threefold difference in rate. J Mol Biol 222: 265-280.
6. Segal E, Fondufe-Mittendorf Y, Chen L, Thastrom A, Field Y, et al. (2006) A genomic code for nucleosome positioning. Nature.
7. Man O, Pilpel Y (2007) Differential translation efficiency of orthologous genes is involved in phenotypic divergence of yeast species. Nat Genet 39: 415-421.
8. Tuller T, Kupiec M, Ruppin E (2007) Determinants of protein abundance and translation efficiency in S. cerevisiae. PLoS Comput Biol 3: e248.
9. Waldman YY, Tuller T, Keinan A, Sharan R, Ruppin E (2010 submitted) Selection for translation efficicncy in human SNPs.
