# Supplementary material for: Genome-Scale Analysis of Translation Elongation with a Ribosome Flow Model
Source: PLoS Comput Biol. 2011 Sep 1;7(9):e1002127. doi: 10.1371/journal.pcbi.1002127 (PMC3164701; doi:10.1371/journal.pcbi.1002127)
Supplement: Text S2 — Endogenous genes in S. cerevisiae , S. pombe , and E. coli : correlation of the predicted rates with protein abundance given mRNA levels and the correlation of the predicted rate multiplies by the mRNA levels with protein abundance. (PDF) [file pcbi.1002127.s029.pdf]

Text S2: Endogenous genes in *S. cerevisiae, S. pombe,* and *E. coli*: correlation of the predicted rates with protein abundance given mRNA levels and the correlation of the predicted rate multiplies by the mRNA levels with protein abundance

E. coli - The correlation with for PA given mRNA: $R = 0.33$ ($p = 1.84*10^{-11}$) for the *RFM vs.* $R = 0.29$ ($p = 3.95*10^{-9}$; 398 genes with PA & mRNA; see Figure S21) for the *tAI*; the correlation of (Rate * mRNA) with PA ($r= 0.71$ for the *RFM vs.* $r = 0.7$ for the *tAI*, $p < 10^{-16}$ in both cases; see Figure S22);

S. pombe - The correlation with PA given mRNA was also higher for the *RFM*, $R=0.39$ ($p < 10^{-16}$) *vs.* 0.307 for the *tAI* ($p < 10^{-16}$) for the tAI (see Figure S23). The correlation of (Rate * mRNA) with PA ($r= 0.7$ for the *RFM* vs. $r = 0.627$ for the *tAI*, $p < 10^{-16}$ in both cases ;see Figure S24);

S. cerevisiae – The correlation with for PA given mRNA: $R = 0.32$ for the *RFM* vs. 0.35 for the *tAI* ($p < 10^{-16}$ in both cases; see Figure S25). The correlation of (Rate * mRNA) with PA: 0.58 for the *RFM vs.* 0.58 for the *tAI* ($p < 10^{-16}$ in both cases; see Figure S26). The correlation with PA $R= 0.49$ for the *RFM vs.* 0.57 for the *tAI* ($p < 10^{-16}$ in both cases).
