# Supplementary material for: Genome-Scale Analysis of Translation Elongation with a Ribosome Flow Model
Source: PLoS Comput Biol. 2011 Sep 1;7(9):e1002127. doi: 10.1371/journal.pcbi.1002127 (PMC3164701; doi:10.1371/journal.pcbi.1002127)
Supplement: Text S3 — The predictions of the tAI and translation efficiency profiles of genes. (PDF) [file pcbi.1002127.s030.pdf]

Text S3: The predictions of the tAI and translation efficiency profiles of genes

The *tAI* is one of the best known codon bias based predictor of protein abundance (see, for example, [1,2,3], r = 0.65 between tAI and protein abundance in *S. cerevisiae*). This measure is the mean co-adaptation of the gene's codon to the tRNA pool of an organism *without considering the order of the codons*. It may sound a bit surprising that such a simple model gives such good performances. In this subsection we will give some explanations for this phenomenon.

One possible explanation is the fact that initiation rate of genes is relatively low. As we have shown, in this regime there are no interactions between ribosomes and the mean "nominal" velocity [4] is good approximation of the actual one.

An alternative or additional explanation for this phenomenon may be the fact that genes (especially highly expressed genes) tend to have a specific design. It was shown that such genes have a non-decreasing profile of translation rate: they start with a region of slower translation rate (30-50 codons); the translation rate afterwards is higher, relatively constant, and usually proportional to the slower beginning ([4]; Figure S27). Such a profile improves the production rate of proteins (the number of proteins per ribosome per time unit, see [4]) and prevent "traffic jams", thus the tAI becomes a good enough predictor. It is possible that that in this regime the tAI performs well (although it is a non-physical and non-causal predictor) simply since the mean speed is a indeed a good approximation of the production rate in this regime (due to the reasons mentioned above).

As we demonstrate in the third section, the tAI performs well mainly for genes that are highly expressed. These genes tend to have the design that appears in Figure S28 but they also have higher ribosome density (*i.e.* higher initiation rate, the correlation between ribosome density [5] and PA is 0.3717 ($p = 7.82 \times 10^{-118}$)). This point suggests that the second explanation is the right one. An additional explanation appears in the main text (robustness to permutations of highly expressed genes).

1. dos Reis M, Savva R, Wernisch L (2004) Solving the riddle of codon usage preferences: a test for translational selection. Nucleic Acids Res 32: 5036-5044.
2. Tuller T, Kupiec M, Ruppin E (2007) Determinants of protein abundance and translation efficiency in S. cerevisiae. PLoS Comput Biol 3: e248.
3. Man O, Pilpel Y (2007) Differential translation efficiency of orthologous genes is involved in phenotypic divergence of yeast species. Nat Genet 39: 415-421.
4. Tuller T, Carmi A, Vestsigian K, Navon S, Dorfan Y, et al. (2010) An evolutionarily conserved mechanism for controlling the efficiency of protein translation. Cell 141: 344-354.
5. Arava Y, Wang Y, Storey JD, Liu CL, Brown PO, et al. (2003) Genome-wide analysis of mRNA translation profiles in Saccharomyces cerevisiae. Proc Natl Acad Sci U S A 100: 3889-3894.
