# Supplementary material for: Genome-Scale Analysis of Translation Elongation with a Ribosome Flow Model
Source: PLoS Comput Biol. 2011 Sep 1;7(9):e1002127. doi: 10.1371/journal.pcbi.1002127 (PMC3164701; doi:10.1371/journal.pcbi.1002127)
Supplement: Text S4 — The genomic rate of abortion of ribosomes has power law decay. (PDF) [file pcbi.1002127.s031.pdf]

Text S4: The genomic rate of *abortion* of ribosomes has power law decay

Measured ribosome density profile appears linear in a log-log graph. Namelythe in ribosomal density in the x site is $C_1 \cdot x^{-\alpha_{real}}$ where α_{real} = 0.158 (Figure 4B); our model also predicts a linear line in a log-log graph but with a smaller slope: the genomic ribosomal density in the x site is $C_2 \cdot x^{-\alpha_{RFM}}$ where α_{RFM} = 0.1 (Figure 4B). These results may suggest that an additional factor $C_2 \cdot x^{-\alpha_{ABR}}$ with α_{ABR} = 0.058 should be added to represent other phenomena such as the genomic rate of *abortion* of ribosomes.
