# Supplementary material for: Genome-Scale Analysis of Translation Elongation with a Ribosome Flow Model
Source: PLoS Comput Biol. 2011 Sep 1;7(9):e1002127. doi: 10.1371/journal.pcbi.1002127 (PMC3164701; doi:10.1371/journal.pcbi.1002127)
Supplement: Text S5 — The initiation rates used in this study are robust and not over-fitted. (PDF) [file pcbi.1002127.s032.pdf]

Text S5: The initiation rates used in this study are robust and not over-fitted

To verify the robustness of the initiation rate use in the analysis of heterologous genes we divided each of the datasets to two parts (each with 50% of the genes). In each case we verified that the same initiation rate optimizes the correlation with PA in both parts.
