# Supplementary material for: Genome-Scale Analysis of Translation Elongation with a Ribosome Flow Model
Source: PLoS Comput Biol. 2011 Sep 1;7(9):e1002127. doi: 10.1371/journal.pcbi.1002127 (PMC3164701; doi:10.1371/journal.pcbi.1002127)
Supplement: Text S6 — Tissue-specific translation rates in Human. (PDF) [file pcbi.1002127.s033.pdf]

Text S6: Tissue-specific translation rates in Human

In humans and other mammals the correlations between expression levels and codon bias are relativly low [1,2,3]. Thus, in the next stage, we aimed at studying how well the predictions of the *RFM* correlate with the expression levels in human tissues, comparing it with the correlations obtained using the *tAI*. The correlations with gene expression of 12,173 genes across 73 tissue are depicted in Figure 6C. As can be seen, some of the correlations with the *RFM* are more than 8 times higher than the correlations with the *tAI* the mean improvement in percentages of the *RFM* with respect to the *tAI* was 80% (Figure 6C, inset). In addition, some of the correlations between tissue-specifc gene expression and *tAI* are negative while in the case of the *RFM all* the correlations are positive. Furthermore, in 71% of the tissues the *RFM* performed better (Figure 6C).

These results demonstrate that our model should be more useful than alternative models for analyzing human gene translation. Thus, it should be helpful for analyzing mutations (see, for example [4]) or SNPs (see, for example, [5,6]) that cause diseases due to problems in gene translation.

1. Waldman YY, Tuller T, Shlomi T, Sharan R, Ruppin E Translation efficiency in humans: tissue specificity, global optimization and differences between developmental stages. Nucleic Acids Res 38: 2964-2974.
2. Urrutia AO, Hurst LD (2001) Codon usage bias covaries with expression breadth and the rate of synonymous evolution in humans, but this is not evidence for selection. Genetics 159: 1191-1199.
3. Chamary JV, Parmley JL, Hurst LD (2006) Hearing silence: non-neutral evolution at synonymous sites in mammals. Nat Rev Genet 7: 98-108.
4. Waldman YY, Tuller T, Sharan R, Ruppin E (2009) TP53 cancerous mutations exhibit selection for translation efficiency. Cancer Res 69: 8807-8813.
5. Kimchi-Sarfaty C, Oh JM, Kim I-W, Sauna ZE, Calcagno AM, et al. (2007) A "Silent" Polymorphism in the MDR1 Gene Changes Substrate Specificity. Science 315: 525-528.
6. Comeron JM (2004) Selective and mutational patterns associated with gene expression in humans: influences on synonymous composition and intron presence. Genetics 167: 1293-1304.
